# Preclinical Characterization of SDFZ‐8, a Highly Potent HDAC1 Inhibitor, for Cancer Immunotherapy

**DOI:** 10.1002/mco2.70500

**Published:** 2025-11-18

**Authors:** Yi Zhou, Jintong Du, Xue Li, Huajun Zhao, Junxin Xue, Yuchen Liu, Xinying Yang, Jinming Yu, Xuben Hou, Hao Fang

**Affiliations:** ^1^ Department of Medicinal Chemistry State Key Laboratory of Discovery and Utilization of Functional Components in Traditional Chinese Medicine Shandong Key Laboratory of Druggability Optimization and Evaluation for Lead Compounds School of Pharmaceutical Science, Cheeloo College of Medicine, Shandong University Ji'nan Shandong China; ^2^ Department of Pharmacy Shandong Provincial Hospital Affiliated to Shandong First Medical University Ji'nan Shandong China; ^3^ Shandong Cancer Hospital and Institute Shandong First Medical University Ji'nan Shandong China; ^4^ Department of Immunopharmaceutical Sciences School of Pharmaceutical Science Cheeloo College of Medicine, Shandong University Ji'nan Shandong China

**Keywords:** cancer immunotherapy, HDAC inhibitor, PD‐L1 blockade, structure‐based drug design

## Abstract

The histone deacetylases (HDACs) family plays a critical role in tumorigenesis and has been identified as having a significant impact on tumor immunity. Herein, we employed a fragment‐centric structure‐based design platform, leading to the discovery of **SDFZ‐8** as a highly potent HDAC1 inhibitor (IC_50_ = 0.4 nM). **SDFZ‐8** exhibits strong antiproliferative effects by enabling histone acetylation and inducing cell apoptosis. Crucially, **SDFZ‐8** treatment led to a significant enhancement of antitumor immunity, as evidenced by increased activation of T cells, enhanced polarization of M1‐type macrophages, improved antigen presentation, and alleviation of the immunosuppressive tumor microenvironment. Specifically, we observed that **SDFZ‐8** notably upregulated the expression of PD‐L1 in both tumor cells and tumor‐infiltrating lymphocytes, which is closely associated with its inhibition against HDAC1. Of particular interest, combining **SDFZ‐8** with PD‐L1 blockade resulted in a synergistic antitumor effect surpassing that of either monotherapy. Taken together, our findings establish **SDFZ‐8** as a novel HDAC1 inhibitor that concurrently targets tumor cells and immune evasion mechanisms, providing a rational combinatorial strategy to enhance cancer immunotherapy efficacy.

## Graphical abstract

In this study, we designed a novel quinoline derivative as a potent HDAC inhibitor **SDFZ‐8** and assessed its capability to enhance cancer immunotherapy, along with its potential synergistic effects with PD‐L1 blockade.

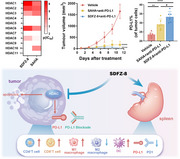

## 1 Introduction

Histone acetylation is one of the most studied epigenetic modifications and plays a critical role in the modification of the chromatin structure [1]. Typically, the acetylation status of histones is mainly regulated by two groups of enzymes, histone deacetylases (HDACs) and histone acetyltransferases [2]. HDACs catalyzed the removal of the acetyl group from the lysine of histone and nonhistone proteins, controlling the elasticity of histone and DNA or other cell signal transduction, thereby stimulating the gene expressions [3, 4, 5]. Based on the dependency of specific cofactors, 18 human HDACs can be divided into two families: the zinc‐dependent HDAC (HDAC1–11) and the NAD^+^‐dependent sirtuin family (SIRT1–7) [6]. Zinc‐dependent HDACs can be subdivided into four classes (class I–IV) based on sequence similarity to yeast deacetylases, among which class I HDACs (HDAC1, HDAC2, HDAC3, and HDAC8) are well studied and proved to be closely related to the development of cancer [7, 8].

Class I HDACs drive tumor progression through dual mechanisms. First, they directly regulate oncogenic pathways by deacetylating transcription factors (e.g., p53, STAT3) and chromatin modifiers [9, 10, 11, 12, 13, 14]. Second, emerging evidence reveals their critical role in shaping the tumor microenvironment (TME)—HDAC1/2 suppress antigen presentation machinery in cancer cells, while HDAC3 promotes immunosuppressive macrophage polarization. These findings position class I HDACs as dual therapeutic targets for simultaneously attacking cancer cells and reversing immune evasion [15, 16, 17, 18, 19, 20].

The development of HDAC inhibitors has evolved significantly since the approval of first‐generation pan‐inhibitors like **Vorinostat** (**SAHA**) and **Romidepsin** for T‐cell lymphomas. While these broad‐spectrum agents demonstrated clinical efficacy, their dose‐limiting toxicities stemming from nonselective HDAC inhibition spurred the pursuit of isoform‐selective compounds [21]. Recent advances have yielded class I‐selective inhibitors such as **Chidamide** (approved in China for peripheral T‐cell lymphoma) and **Mocetinostat** (HDAC1/2/3/11 selective inhibitor). However, challenges persist in balancing selectivity, potency, and pharmacokinetic properties, particularly for solid tumors where poor vascularization limits drug accumulation [22]. Heretofore, none of existing HDAC inhibitor has demonstrated clinically meaningful synergy with immune checkpoint blockers, likely due to incomplete understanding of HDAC–immune crosstalk [23].

To address these limitations, we hypothesized that a class I‐selective HDAC1 inhibitor with optimized pharmacokinetics could achieve dual tumor‐intrinsic and immune‐modulatory effects. Our strategy leveraged the *AlphaSpace* platform—a fragment‐centric computational tool that maps druggable subpockets in HDAC1's catalytic domain. Through iterative structure‐based design, we developed **SDFZ‐8**, a quinoline derivative engineered for enhanced isoform selectivity and TME penetration.

In this study, we report the novel HDAC1 inhibitor, **SDFZ‐8**, developed through structure‐based design using our fragment‐centric *AlphaSpace* platform [24]. Unlike existing pan‐HDAC inhibitors (e.g., **SAHA**), **SDFZ‐8** achieves subnanomolar HDAC1 potency (IC_50_ = 0.4 nM, 92‐fold superior to **SAHA**) while maintaining class I selectivity, addressing a critical limitation of current therapies that suffer from off‐target toxicity. Beyond direct tumor suppression, our work uncovers a novel dual mechanism: **SDFZ‐8** not only inhibits HDAC1‐driven oncogenesis but also reprograms the tumor immune microenvironment by activating T cells, enhancing antigen presentation, and reversing macrophage‐mediated immunosuppression. Importantly, we establish a previously unrecognized link between HDAC1 inhibition and PD‐L1 upregulation, providing a rational basis for combining **SDFZ‐8** with PD‐L1 blockade. This study bridges a key gap in HDAC‐targeted therapy by demonstrating that precision HDAC1 inhibition can simultaneously target cancer cells and overcome immune evasion, offering a transformative paradigm for small‐molecule cancer immunotherapy.

## 2 Results

### 2.1 Rational Design of Quinoline Derivatives for HDAC Inhibition

**FIGURE 1:**
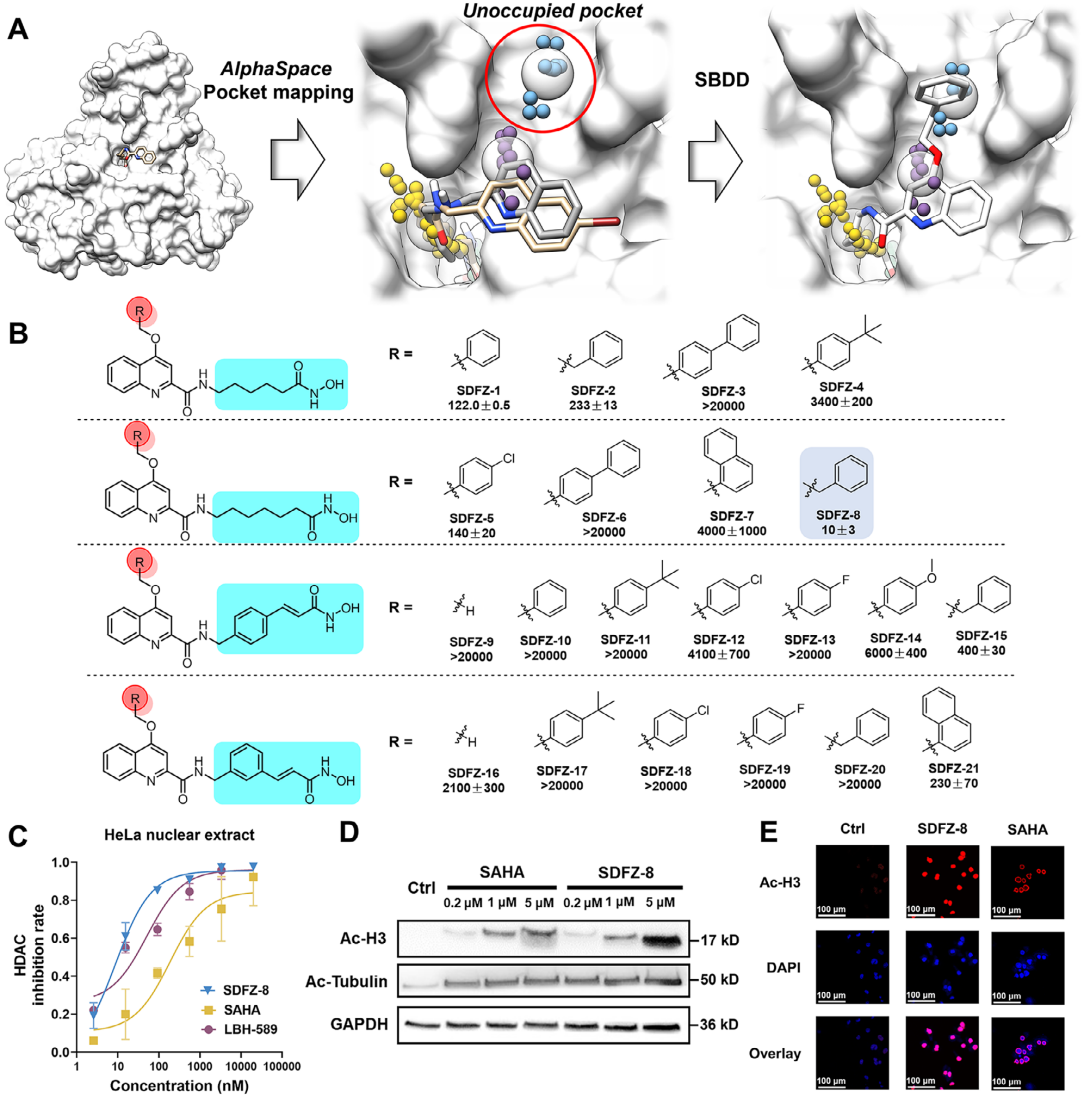
Rational design and in vitro HDAC inhibitory profiling of **SDFZ‐8**. (A) Structure‐based design of new HDAC inhibitors using fragment‐centric topographic mapping strategy. The favorable binding mode of **SDFZ‐8** was predicted using Glide. The lead compounds **WL‐9w** and **CC‐4a** were presented as tan and gray molecules in the active site of HDAC1 (PDB: 5ICN). The transparent spheres represent pockets that are detected by *AlphaSpace*. The alpha‐atoms clusters in each pocket were presented as small spheres with different colors. The unoccupied pockets were marked with a red cycle. The images were illustrated by UCSF Chimera. (B) The chemical structure and HDAC inhibitory activities of the quinoline derivatives were determined by HeLa extract. Results are expressed as the mean ± SD of three separate determinations. (C) The HDAC inhibition curve of selected compounds at different concentrations of three separate determinations. (D) Western blotting analysis of MDA‐MB‐231 cells incubated with selected compounds (µM) for 24 h. (E) Immunofluorescence of ac‐H3 in MDA‐MB‐231 cells incubated with 1 µM selected compounds for 24 h.

Quinoline is widely utilized in drug design and has been considered as a privileged scaffold in drug development [25]. Our previous research efforts have yielded a variety of quinoline derivatives as HDAC inhibitors (e.g., **CC‐4a** and **WL‐9w**). However, there is still a need to enhance their potency and selectivity [26, 27]. A major challenge in developing selective HDAC inhibitors lies in the high degree of structural conservation among different HDAC subtypes. To address this challenge, we utilized *AlphaSpace* [28] to detect the binding pockets of our lead compounds in HDAC1 and identified unoccupied pocket the protein surfaces surrounding the 4‐position of quinoline group (Figure 1A). Based on these findings, we undertook structural optimization of lead compounds by introducing various substitutions at the 4‐position of quinoline and by directly attaching the amide linkage to the quinoline (Figure 1B). The HDAC inhibitory activities of all target compounds were initially assessed using HeLa nuclear extract, which mainly consist class I HDACs. The known HDAC inhibitors **SAHA** and **LBH‐589** were used as positive controls for comparison (Figure S1). As indicated in Figure 1B, the long‐chain hydroxamic acids exhibited superior HDAC inhibitory activity. Specifically, hydroxamic acids with a chain length of 6 carbons demonstrated higher activity compared with those with a chain length of 5 carbons (**SDFZ‐8 **vs. **SDFZ‐2**). Additionally, for the R group, benzyl was found to be more favorable than substituted phenyl or other groups (**SDFZ‐8** vs. **SDFZ‐5**, **SDFZ‐6**, and **SDFZ‐7**). The target compound **SDFZ‐8** exhibits nanomolar HDAC inhibitory activity (IC_50_ = 10 nM), surpassing the positive control drugs **SAHA** by 28.6‐fold and **LBH‐589** by 2.5‐fold (Figure 1C). However, for *N*‐hydroxycinnamamide target compounds, both meta‐ and para‐substituted cinnamamide derivatives generally displayed poor activity (**SDFZ‐9**–**SDFZ‐21**), suggesting that *N*‐hydroxycinnamamide derivatives may not align with our design objectives.

### 2.2 HDAC Isoform Selectivity of SDFZ‐8

Since **SDFZ‐8** emerges as a potent class I HDAC inhibitor, we further assessed its isoform selectivity against different HDACs (Table 1). Specifically, among class I HDACs, **SDFZ‐8** demonstrated high potent inhibition of HDAC1 (IC_50_ = 0.4 nM) and HDAC2 (IC_50_ = 5.8 nM), along with more than 800‐fold selectivity toward HDAC3 and HDAC8 (Figure S2). Within class IIb HDACs, **SDFZ‐8** displayed potent inhibition against HDAC6 (IC_50_ = 3.1 nM) and demonstrated high selectivity toward HDAC10 (IC_50_ > 10,000 nM). Additionally, **SDFZ‐8** also exhibited high selectivity against class IIa HDACs (HDAC4, HDAC5, HDAC7, and HDAC9) and class IV HDAC (HDAC11). These results suggest that **SDFZ‐8** represents a highly potent HDAC1/2/6 inhibitor with improved isoform selectivity compared with **SAHA**.

**TABLE 1:** HDAC isoform selectivity of **SDFZ‐8** and **SAHA**.

HDAC isozymes		IC_50_ values (nM)^a^		
		SDFZ‐8	SAHA
Class I	HDAC1	0.4 ± 0.1	37 ± 7
	HDAC2	5.8 ± 1.5	15.2 ± 1.9
	HDAC3	300 ± 100	45 ± 20
	HDAC8	750 ± 70	6400 ± 600
Class IIa	HDAC4	>10,000	>10,000
	HDAC5	>10,000	>10,000
	HDAC7	>10,000	>10,000
	HDAC9	>10,000	>10,000
Class IIb	HDAC6	3.1 ± 0.5	90 ± 7
	HDAC10	>10,000	100 ± 40
Class IV	HDAC11	>10,000	>10,000

^a^ Results are expressed as the mean ± SD of three separate determinations.

### 2.3 SDFZ‐8 Stimulates the Acetylation of Histone H3 and α‐Tubulin

To confirm the HDAC inhibitory effect of **SDFZ‐8** at the cellular level, western blotting was conducted on MDA‐MB‐231 cells. As illustrated in Figure 1D, compound **SDFZ‐8** and **SAHA** both displayed an upregulation in the level of acetylated histone H3 (Ac‐H3), a key substrate of class I HDACs. Moreover, **SDFZ‐8** and **SAHA** exhibited an upregulation in the level of acetylated tubulin (Ac‐tubulin), which is a substrate of HDAC6. These results indicated that **SDFZ‐8** has the capability to inhibit class I HDACs (HDAC1, HDAC2, and HDAC3) as well as HDAC6 in MDA‐MB‐231 cells. In addition, we conducted immunofluorescence experiments to further investigate the accumulation of ac‐H3. As depicted in Figure 1E, the immunofluorescence intensities of ac‐H3 in MDA‐MB‐231 cells treated with **SDFZ‐8** were notably stronger than those of cells treated with **SAHA**, consistent with the immunoblotting results. To further assess the cellular distribution of ac‐H3, we analyzed the colocalization of ac‐H3 and DAPI (a nuclear dye). The Pearson's *R* value and the overlap *R* value of ac‐H3 immunofluorescence and DAPI were determined to be 0.86 and 0.88, indicating a strong correlation between ac‐H3 and the nucleus (Figure S3).

### 2.4 In Vitro Antiproliferative Activities of SDFZ‐8

**FIGURE 2:**
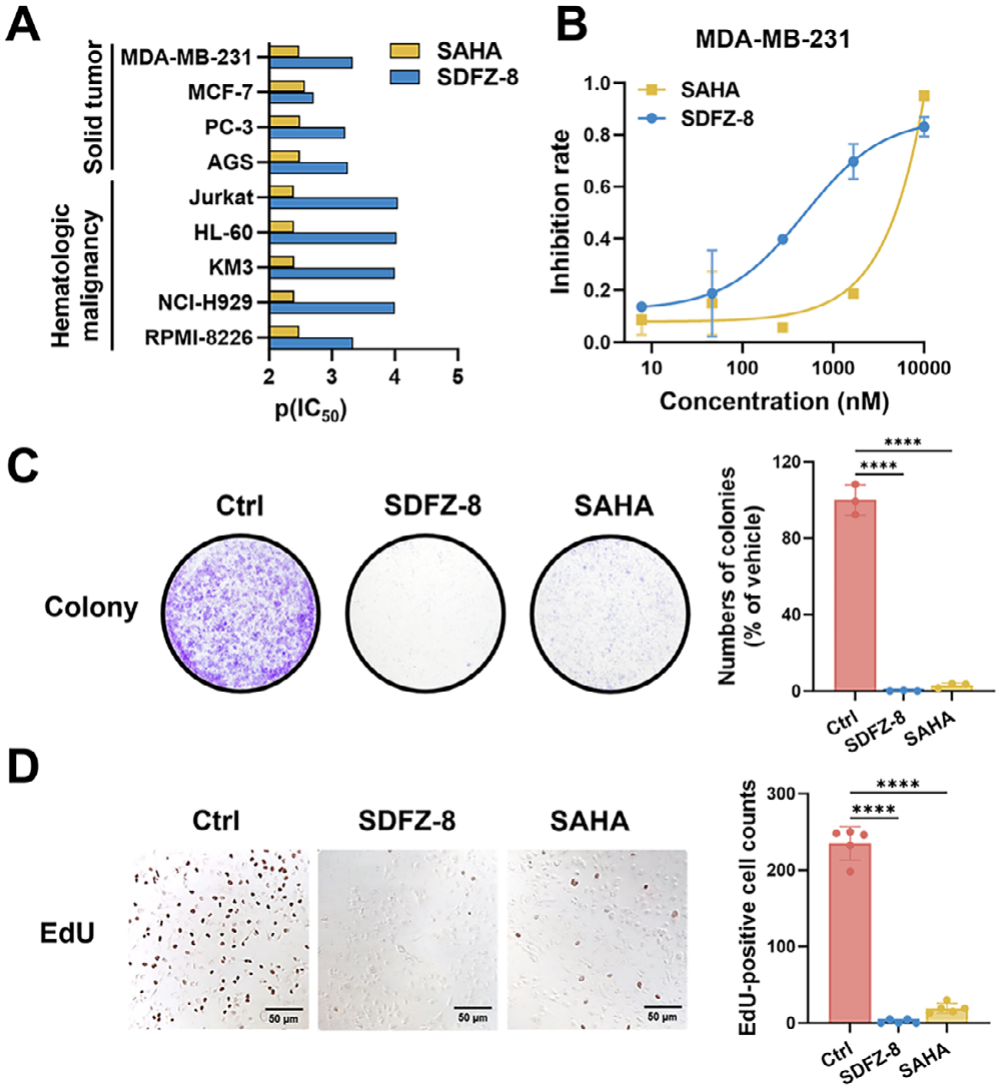
**SDFZ‐8** exhibits potent antiproliferative effects across cancer cell lines. (A) The antiproliferative activity of **SDFZ‐8** and SAHA against different tumor cell lines after 48 h treatment. The pIC_50_ value was calculated as the negative logarithm of the IC_50_ in molar concentration. (B) The dose‐dependent inhibition curve of **SDFZ‐8** and **SAHA** on the cell viability of MDA‐MB‐231 cell of three separate determinations. (C) The clonogenic ability of MDA‐MB‐231 cells after treatment with 2 µM **SDFZ‐8** and 2 µM **SAHA** for 48 h. (D) The EdU staining results of MDA‐MB‐231 cells incubated with 2 µM **SDFZ‐8** and 2 µM **SAHA** for 24 h.

Nine different human cancer cell lines were selected, including multiple myeloma cells RPMI‐8226, NCI‐H929, and KM3, acute promyelocytic leukemia cells HL‐60, T lymphocytic leukemia cells Jurkat, as well as solid tumor cell lines AGS, MDA‐MB‐231, MCF‐7, and PC‐3, to assess the antiproliferative potencies of **SDFZ‐8** (Table S1 and Figure S4). As shown in Figure 2A, **SDFZ‐8** exhibited superior antiproliferative activity (threefold to ninefold) compared with **SAHA**. It is worth noting that the limited efficacy of approved HDAC inhibitors against solid tumor cells as compared with hematologic neoplasm cells has constrained their clinical application [29]. As depicted in Figure 2A, our newly designed compound **SDFZ‐8** demonstrated enhanced antiproliferative activity against solid tumor cells, including AGS, PC‐3, MDA‐MB‐231, and MCF‐7 cells. Triple‐negative breast cancer cells (MDA‐MB‐231) represent one of the most resistant types of breast cancer. Notably, **SDFZ‐8** exhibited threefold better antiproliferation activity than **SAHA** in MDA‐MB‐231 cells (Figure 2B). To further explore antiproliferative activities of **SDFZ‐8**, the colony formation assay and EdU assay were performed. As shown in Figure 2C, **SDFZ‐8** significantly inhibited the colony formation of MDA‐MB‐231. Besides, **SDFZ‐8** also decreased the EdU positive cells (Figure 2D). The results confirmed the antiproliferative potencies of **SDFZ‐8**.

### 2.5 Effect of SDFZ‐8 on Cell Apoptosis

**FIGURE 3:**
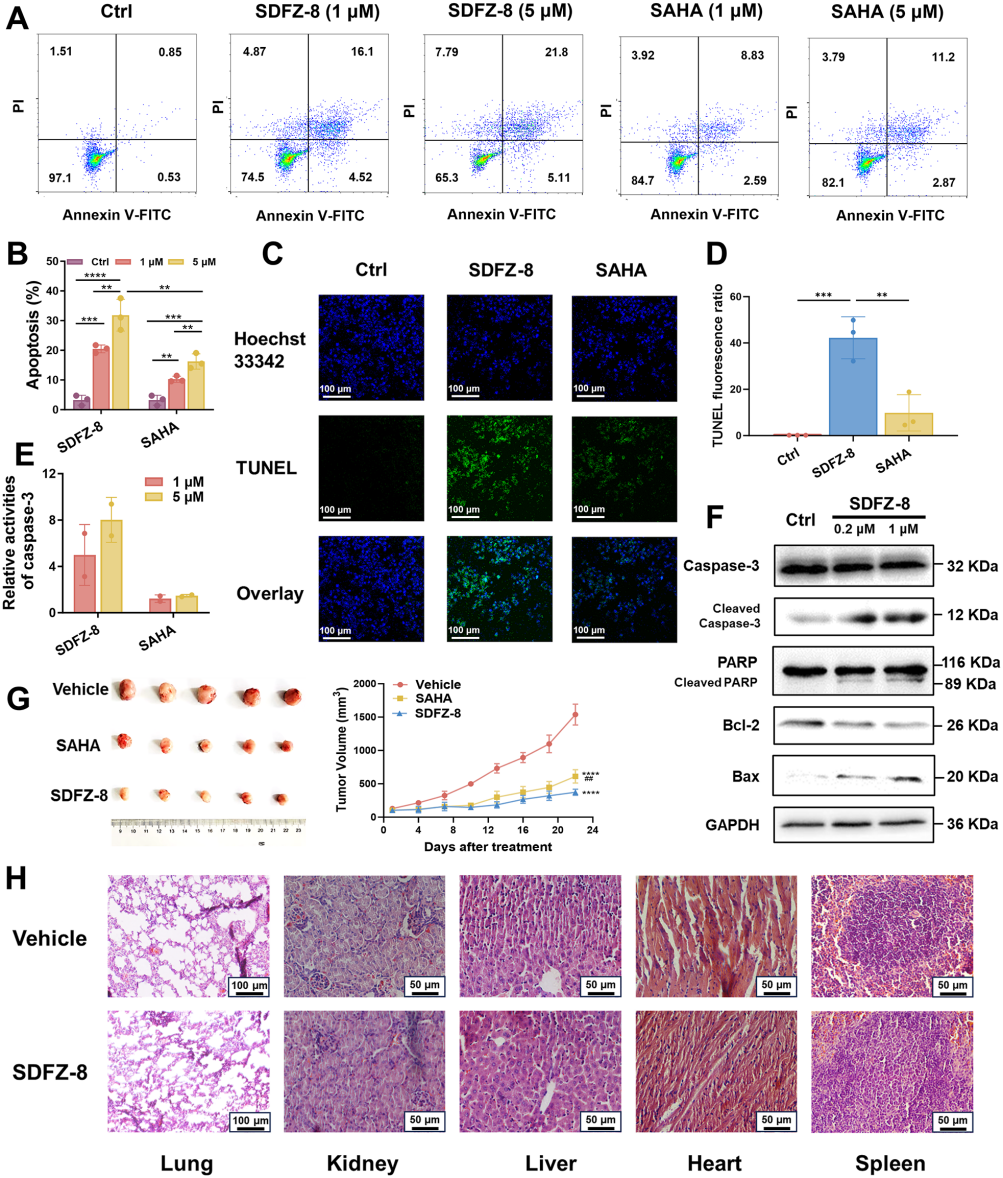
**SDFZ‐8** induces apoptosis and suppresses tumor growth in vivo. (A) Annexin V‐FITC/PI dual dying results of MDA‐MB‐231 cells incubated with selected compounds for 24 h. (B) Statistical analysis of apoptosis induced in MDA‐MB‐231 by selected compounds in Annexin V‐FITC/PI assay. (C) TUNEL dying results of MDA‐MB‐231 incubated with 5 µM selected compounds for 24 h. (D) Statistical analysis of apoptosis induced in MDA‐MB‐231 by selected compounds in TUNEL assay. (E) Caspase‐3 activation of MDA‐MB‐231 cells after treatment with indicated compounds for 24 h of three separate determinations detected by GreenNuc assay. (F) The levels of Bcl‐2, Bax, Caspase‐3/cleaved‐Caspase‐3 and PARP1/cleaved‐PARP1 in MDA‐MB‐231 cells after **SDFZ‐8** treatment for 24 h. (G) In vivo tumor growth inhibition of **SDFZ‐8** in the MDA‐MB‐231 xenograft model (*n* = 3). (H) Hematoxylin–eosin staining results of the lung, kidney, liver, heart and spleen. (****p < 0.0001 vs. vehicle; ##*p* < 0.01 vs. **SDFZ‐8**).

We examined the effects of **SDFZ‐8** on the apoptosis using the Annexin V‐FITC/PI method, which quantitatively determines the levels of apoptotic cells. As illustrated in Figure 3A,B, the apoptosis promotion induced by **SDFZ‐8** was found to be superior to that of **SAHA**. Meanwhile, the TdT‐mediated dUTP nick‐end labeling (TUNEL) assay was also utilized to further assess the effects of HDAC inhibitors on cell apoptosis. In brief, MDA‐MB‐231 cells were exposed to different HDAC inhibitors for 24 h, stained with the FITC‐TUNEL kit, and imaged using a confocal laser scanning microscope. As demonstrated in Figure 3C,D, the treatment with **SDFZ‐8** led to a higher intensity of green fluorescence compared with **SAHA**. We utilized the GreenNuc assay to determine the level of caspase‐3 activation, which is an essential hallmark of cell apoptosis, in MDA‐MB‐231 cells. As depicted in Figure 3E, **SDFZ‐8** induced caspase‐3 activation in a dose‐dependent manner, which is significantly higher than that observed for **SAHA**.

Given the established roles of the Bcl‐2 family and caspase‐3 in HDAC inhibitor‐induced apoptosis, we examined the expression of relevant proteins. The results demonstrated that **SDFZ‐8** treatment upregulated the proapoptotic protein Bax while downregulating the antiapoptotic protein Bcl‐2 (Figure 3F), suggesting a shift in the cellular balance toward apoptosis. Moreover, we detected an increase in the levels of cleaved caspase‐3 and cleaved PARP, upon **SDFZ‐8** treatment (Figure 3F). These results mentioned above clearly indicate that apoptosis in MDA‐MB‐231 cells was significantly induced by our newly designed HDAC inhibitor.

### 2.6 Preliminary Pharmacokinetic Study of SDFZ‐8

Based on the results of in vitro HDAC inhibition and antiproliferation activity, **SDFZ‐8** was chosen for further pharmacokinetic assessment. In mouse liver microsomes (Figure S5), **SDFZ‐8** displayed improved metabolic stability compared with **SAHA**, characterized by a longer half‐life (*T*_1/2_ = 12.31 min) and a lower intrinsic clearance rate (CL_int(mic)_ = 56.28 µL/min/mg). Subsequently, **SDFZ‐8** was administered intravenously (i.v.) at 2.5 mg/kg or orally (i.g.) at 10 mg/kg using male Sprague–Dawley (SD) rats to evaluate its in vivo pharmacokinetic properties. As shown in Table 2, **SDFZ‐8** demonstrated a satisfactory half‐life (i.v., *T*_1/2_ = 2.29 h; i.g., *T*_1/2_ = 5.74 h), maximum plasma concentration (i.v., *C*_max_ = 1750 ng/mL; i.g., *C*_max_ = 658.8 ng/mL), and drug exposure (i.v., AUC_0−_ *_t_* = 2729 ng/mL h; i.g., AUC_0−_ *_t_* = 3084 ng/mL h). The oral bioavailability of **SDFZ‐8** was determined to be 26.11%, signaling its viability as a suitable dosing route for further in vivo investigations.

**TABLE 2:** In vivo pharmacokinetic parameters of **SDFZ‐8**.

PK parameters	i.v. (2.5 mg/kg)^a^	i.g. (10 mg/kg)^a^
*T*_1/2_ (h)	2.29 ± 1.24	5.74 ± 1.51
*T*_max_ (h)	0.08 ± 0.01	0.33 ± 0.14
*C*_max_ (ng/mL)	1750 ± 498.0	658.8 ± 63.09
AUC_0−_ *_t_* (ng/mL h)	2729 ± 259.8	3084 ± 205.9
AUC_0−_ *_∞_* (ng/mL h)	3121 ± 241.6	3259 ± 662.7
*F*%	—	26.11%

^a^ All experiments are expressed as the mean ± standard deviation of three separate determinations (mean ± SD).

### 2.7 In Vivo Antitumor Activity of SDFZ‐8 in the MDA‐MB‐231 Xenograft Model

To assess the in vivo antitumor efficacy of **SDFZ‐8**, we established a xenograft model in nude mice (BALB/c‐nu) using MDA‐MB‐231 cells. Specifically, 1.1 × 10^7^ MDA‐MB‐231 cells were subcutaneously injected, and when the tumor size reached approximately 100 mm^3^, **SDFZ‐8** (60 mg/kg/day) or **SAHA** (60 mg/kg/day) were administered via oral gavage for 22 days. As illustrated in Figure 3G, both **SDFZ‐8** and **SAHA** significantly suppressed tumor growth compared with the vehicle group (*p* < 0.001). It is worth noting that **SDFZ‐8** (TGI = 76%) demonstrated superior in vivo antitumor activity compared with **SAHA** (TGI = 60%) (**Table** **S2**). Collectively, **SDFZ‐8** effectively suppressed tumor growth in MDA‐MB‐231 xenograft BALB/c‐nu mice without adversely affecting the general health of the mice (Figures 3H and S6A).

### 2.8 SDFZ‐8 Upregulates PD‐L1 Expression by Inhibition of HDAC1

**FIGURE 4:**
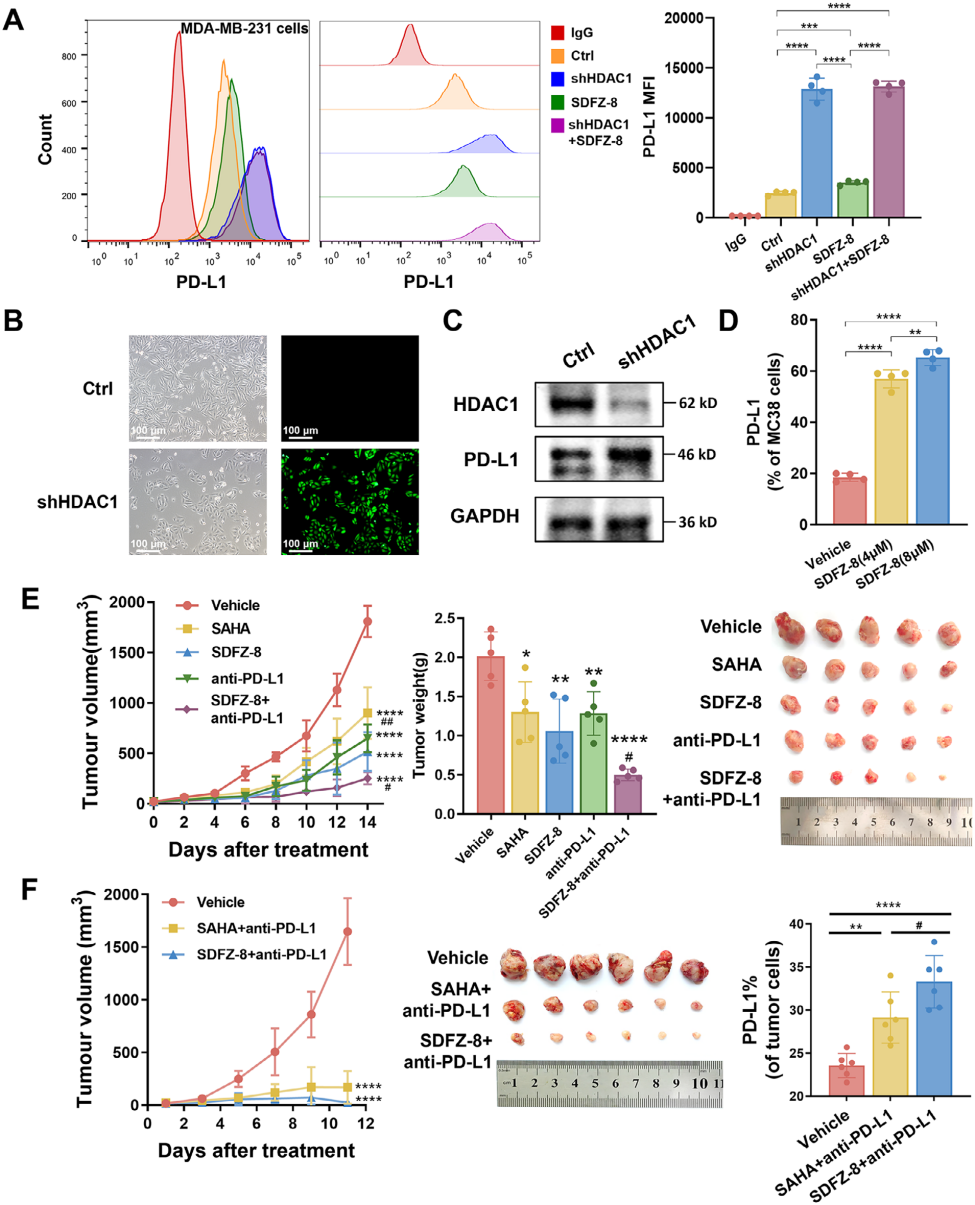
**SDFZ‐8** promotes immune activation and synergizes with anti‐PD‐L1 therapy in the MC38 model. (A–C) Impacts of HDAC1 inhibition on PD‐L1 expression in MDA‐MB‐231 cells and knockdown of HDAC1 using shRNA virus infected for 96 h. (D) Treatment with the HDAC1 inhibitor **SDFZ‐8** for 24 h upregulates PD‐L1 expression in MC38 cells of four separate determinations (*n* = 4/each group). (E) In vivo antitumor potency of **SDFZ‐8** in the MC38 syngeneic model (*n* = 5/each group). (F) Comparison of the tumor growth suppression and PD‐L1 regulation between **SAHA** and **SDFZ‐8** in combination with anti‐PD‐L1 blockade (*n* = 6/each group). (****p < 0.0001, **p < 0.01, *p < 0.05 vs. vehicle; ##*p *< 0.01, #*p *< 0.05 vs. **SDFZ‐8**).

Previous studies have highlighted the essential role of HDACs as epigenetic modulators of tumor immunity and indicated promising potential for cancer immunotherapy with HDAC inhibitors [30, 31, 32, 33, 34]. Specifically, it has been reported that HDAC inhibitors could elevate the expression level of PD‐L1 in melanoma, thereby enhancing immunotherapy with PD‐1 blockade [35]. These findings underscore the potential involvement of HDACs in the regulation of the PD‐1/PD‐L1 signaling pathway. Using flow cytometry, we also observed an increase in PD‐L1 expression following treatment with **SDFZ‐8** in MDA‐MB‐231 cells (Figure 4A). These results further support the potential of **SDFZ‐8** in modulating the PD‐1/PD‐L1 axis with potential implications for cancer immunotherapy. Given the strong evidence supporting **SDFZ‐8** as a potent HDAC1 inhibitor, we pursued further investigation into the role of HDAC1 in stimulating PD‐L1 expression. As depicted in Figure 4A**–C**, knockdown of HDAC1 led to an increase in PD‐L1 expression in MDA‐MB‐231 cells, and treatment of **SDFZ‐8** in HDAC1‐knockdown MDA‐MB‐231 cells did not yield a further increase in PD‐L1 expression. These findings strongly suggest that **SDFZ‐8** has the capacity to upregulate PD‐L1 expression by specifically inhibiting HDAC1, thereby implying the potential for combined use with anti‐PD‐1 or anti‐PD‐L1 blockade therapies.

### 2.9 SDFZ‐8 Augment Tumor Immunity and Synergetic with Anti‐PD‐L1 Blockade in the MC38 Syngeneic Model

Treatment of **SDFZ‐8** also increased the expression level of PD‐L1 in MC38 cells (Figure 4D). To gain further insights into the immune modulation effects of **SDFZ‐8** in vivo, we utilized the MC38 syngeneic C57BL/6J mice model and treated them with **SAHA**, **SDFZ‐8**, anti‐PD‐L1 blockade, and a combination of **SDFZ‐8** and anti‐PD‐L1 blockade. As demonstrated in Figure 4E, the treatment with **SDFZ‐8** led to a significant reduction in tumor volume and tumor weight compared with the vehicle group, thereby confirming the in vivo antitumor efficacy of **SDFZ‐8**. Furthermore, our results indicated that **SDFZ‐8** (TGI = 71%) exhibited greater potency than **SAHA** (TGI = 50%), a finding consistent with the results from the xenograft model. In particular, the combined use of **SDFZ‐8** with anti‐PD‐L1 blockade (atezolizumab) produced more potent antitumor effects (TGI = 86%) than monotherapy (TGI = 64%) (**Table** **S2**). No significant changes in body weight were observed during the administration period (Figure S6B). Furthermore, our hematoxylin–eosin (H&E) staining analysis indicated that **SDFZ‐8** exhibited low toxicity in various organs (heart, liver, spleen, lung, and kidney) and exerted significant antitumor effects in tumor tissue (**Figure** **S7**). The comparison of the antitumor effects of **SDFZ‐8** and **SAHA** in combination with anti‐PD‐L1 blockade revealed that the **SDFZ‐8 **+ anti‐PD‐L1 group displayed superior antitumor activity compared with the **SAHA **+ anti‐PD‐L1 group (Figure 4F). Notably, we also observed higher PD‐L1 expression levels in the **SDFZ‐8 **+ anti‐PD‐L1 group than in the **SAHA **+ anti‐PD‐L1 group.

### 2.10 SDFZ‐8 Stimulates Potent Antitumor Immunity

**FIGURE 5:**
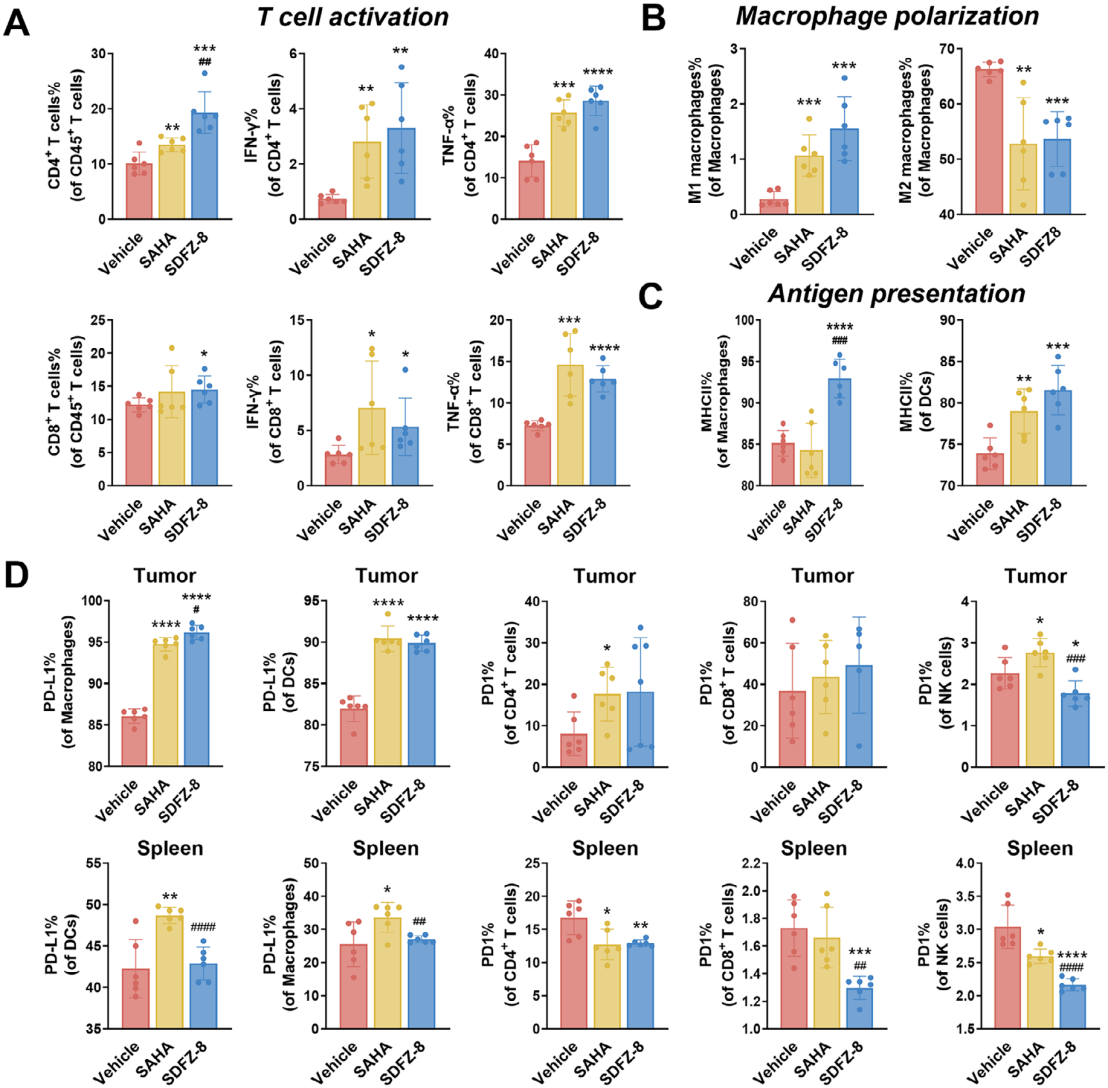
**SDFZ‐8** enhances antitumor immunity through immune cell modulation. (A) Impacts of **SDFZ‐8** on the T cell infiltration and activation of five separate determinations. (B) Impacts of **SDFZ‐8** on macrophage cells in the tumor environment of five separate determinations. (C) Impacts of **SDFZ‐8** on MHC II expression in macrophages and DCs of five separate determinations. (D) Impacts of **SDFZ‐8** on PD‐1 and PD‐L1 expression in tumor‐infiltrating immune cells and spleen immune cells of five separate determinations. *n* = 6/each group. (****p < 0.0001, ***p < 0.001, **p < 0.01, *p < 0.05 vs. vehicle; ####*p* < 0.0001, ###*p* < 0.001, ##*p *< 0.01 vs. **SAHA**).

To assess the impact of **SDFZ‐8** on tumor immunity, we conducted measurements of the composition and functional levels of different immune cells using flow cytometry. Activated T cells play a crucial role in antitumor immunity. As illustrated in Figure 5A, treatment with **SDFZ‐8** resulted in a more significant increase in tumor‐infiltrating lymphocytes (TILs) than **SAHA**, particularly in the populations of CD4^+^ T cells and CD8^+^ T cells. Both **SAHA** and **SDFZ‐8** demonstrated an upregulation in the expression levels of functional molecules associated with T cell activation, including IFN‐γ and TNF‐α, in CD4^+^ T cells and CD8^+^ T cells. Additionally, the proportion of CD4^+^ T cells, CD8^+^ T cells, and NK cells was elevated in the spleen following treatment with **SDFZ‐8**, whereas **SAHA** did not induce such effects (Figure S8). These findings strongly indicate that **SDFZ‐8** enhances antitumor immunity by promoting T cell activation.

On the other hand, tumor‐associated macrophages (TAMs) are among the major tumor‐infiltrating immune cells and are generally categorized into two primary phenotypes, M1 and M2. Previous research suggests that M1 TAMs can suppress cancer progression, while M2 TAMs exhibit the opposite effect [36]. Our analysis, which involved the detection of specific markers for M1‐type macrophages (iNOS [37]) and M2‐type macrophages (CD206^38^), revealed that the percentage of M2 TAMs in TILs was over 65% in the vehicle group, indicating a tumor‐promoting microenvironment (Figure 5B). Notably, both **SDFZ‐8** and **SAHA** robustly promoted M1 polarization and suppressed the M2 polarization of TAMs. Additionally, the recognition and presentation of tumor antigens are crucial for eliciting antitumor immunity. Dendritic cells (DCs) and macrophages are essential antigen‐presenting cells responsible for recognizing and presenting tumor antigens. Our findings illustrated in Figure 5C indicate that treatment with **SDFZ‐8** significantly increased the surface expression of MHC class II (MHC II) in both DCs and macrophages, while **SAHA** only impacted MHC II in DCs.

The results from our in vitro experiments indicating that **SDFZ‐8** upregulates PD‐L1 expression in MC38 cells (Figure 4D) were consistently reflected in the in vivo experiment, where treatment with **SDFZ‐8** led to an upregulation in the expression of PD‐L1 in tumor‐infiltrating immune cells (DCs and macrophages) (Figure 5D). Interestingly, **SDFZ‐8** did not affect PD‐1 expression in tumor‐infiltrating CD4^+^ T cells and CD8^+^ T cells, and downregulated PD‐1 expression in tumor‐infiltrating NK cells. Furthermore, **SDFZ‐8** significantly decreased PD‐L1 and PD‐1 expression in various immune cells in the spleen. In contrast, the pan‐HDAC inhibitor **SAHA** upregulated both PD‐L1 and PD‐1 expression in tumor‐infiltrating immune cells. In the spleen, **SAHA** upregulated PD‐L1 expression in DCs and macrophages and downregulated PD‐1 expression in CD4^+^ T cells and NK cells. Consequently, the nonselective HDAC inhibitor enhanced PD‐1/PD‐L1 signaling and indicated an immunosuppressive role. Our newly designed high‐potency and HDAC1/2/6 selective inhibitor, **SDFZ‐8**, specifically upregulated PD‐L1 expression and downregulated PD‐1 expression in tumor‐infiltrating and spleen immune cells, suggesting its potential to enhance the efficacy of anti‐PD‐L1 blockade without promoting immunosuppression effects mediated by PD‐1/PD‐L1 signaling.

### 2.11 SDFZ‐8 Augment Tumor Immunity and Synergetic with Anti‐PD‐L1 Blockade in the B16F10 Syngeneic Model

**FIGURE 6:**
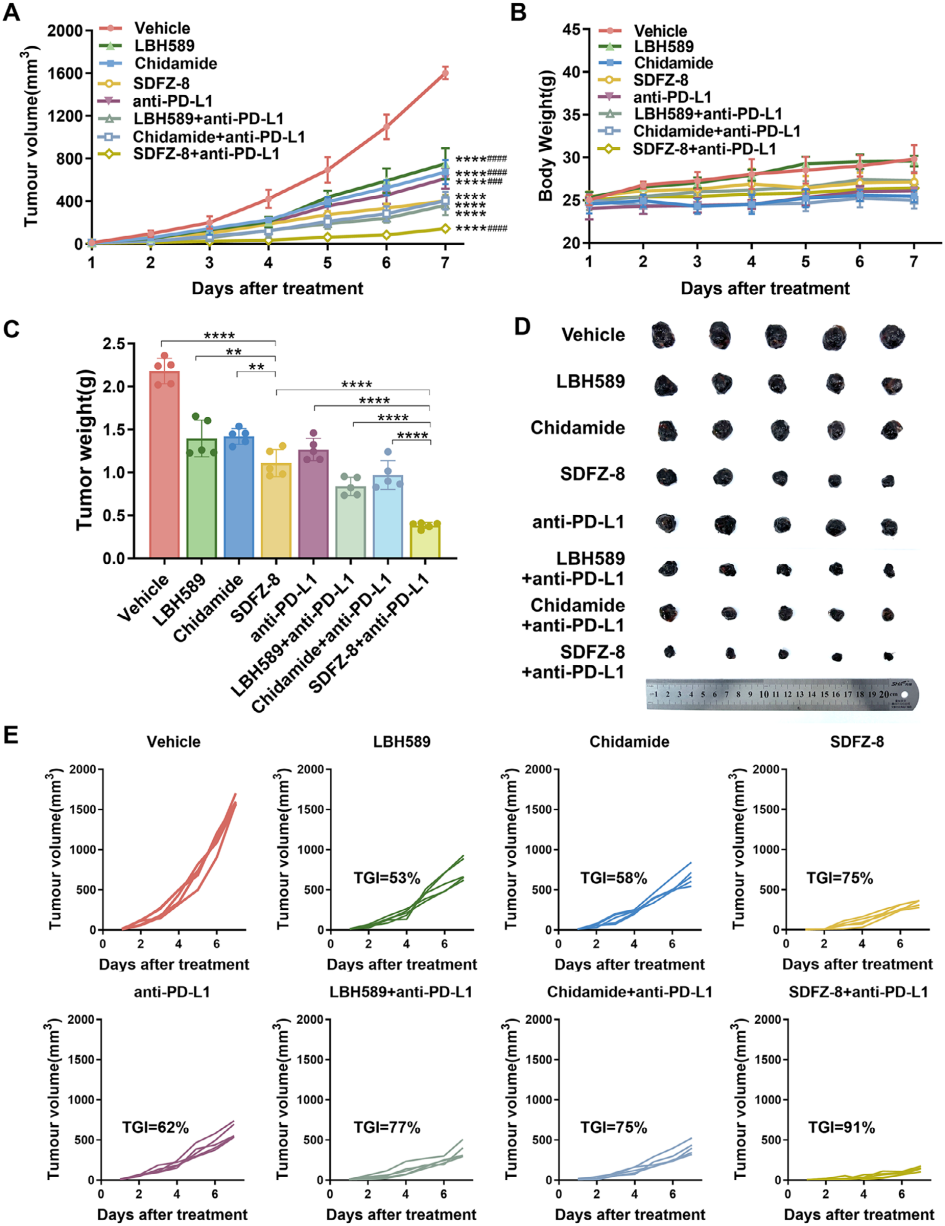
**SDFZ‐8** synergizes with anti‐PD‐L1 to inhibit tumor growth in the B16F10 melanoma model. (A and B) The curves of tumor volumes (A) and body weights (B) of mice treated with indicated compounds combined with or without anti‐PD‐L1 blockade in the B16F10 syngeneic model. (C and D) The weights (C) and representative images (D) of tumors in the B16F10 syngeneic model. (E) The tumor volume curves of each mouse in different groups in the B16F10 syngeneic model. *n* = 5/each group. (****p < 0.0001, **p < 0.01 vs. vehicle; ####*p *< 0.0001, ###*p *< 0.001 vs. **SDFZ‐8**).

To substantiate the therapeutic potential of **SDFZ‐8** across broader cancer types, we conducted validation studies using the B16F10 xenograft melanoma model. As shown in Figure 6A–E and **Table** **S2**, **SDFZ‐8** significantly reduced tumor volume and tumor weight (TGI = 75%) compared with the other single HDAC inhibitor treatment group. Furthermore, the combination of **SDFZ‐8** with anti‐PD‐L1 blockade produced more potent antitumor effects (TGI = 91%) than monotherapy and the combination of other HDAC inhibitors with anti‐PD‐L1 blockade. No significant changes in body weight were observed during the administration period. (Figure S6C) These investigations confirmed the efficacy of **SDFZ‐8** in suppressing melanoma progression and revealed synergistic tumor growth inhibition when administered in combination with anti‐PD‐L1 immunotherapy.

## 3 Discussion

In this study, a new series of quinolone‐based HDAC inhibitors were designed using fragment‐centric pocket analysis. Among them, **SDFZ‐8** was identified as the most active compound, demonstrating potent inhibition of HDAC1/2/6 (IC_50_ = 0.4–5.8 nM) and selective over other HDAC isoforms. Additionally, **SDFZ‐8** exhibited superior antiproliferative activity against various tumor cells compared with the positive control drug SAHA. In MDA‐MB‐231 cells, **SDFZ‐8** promoted acetylation of histone H3 and significantly induced cell apoptosis. Preliminary pharmacokinetic studies indicated liver microsome stability and oral bioavailability of **SDFZ‐8**. Furthermore, **SDFZ‐8** effectively reduced tumor burden in both the MDA‐MB‐231 xenograft model and the MC38 syngeneic model, surpassing the performance of SAHA.

**FIGURE 7:**
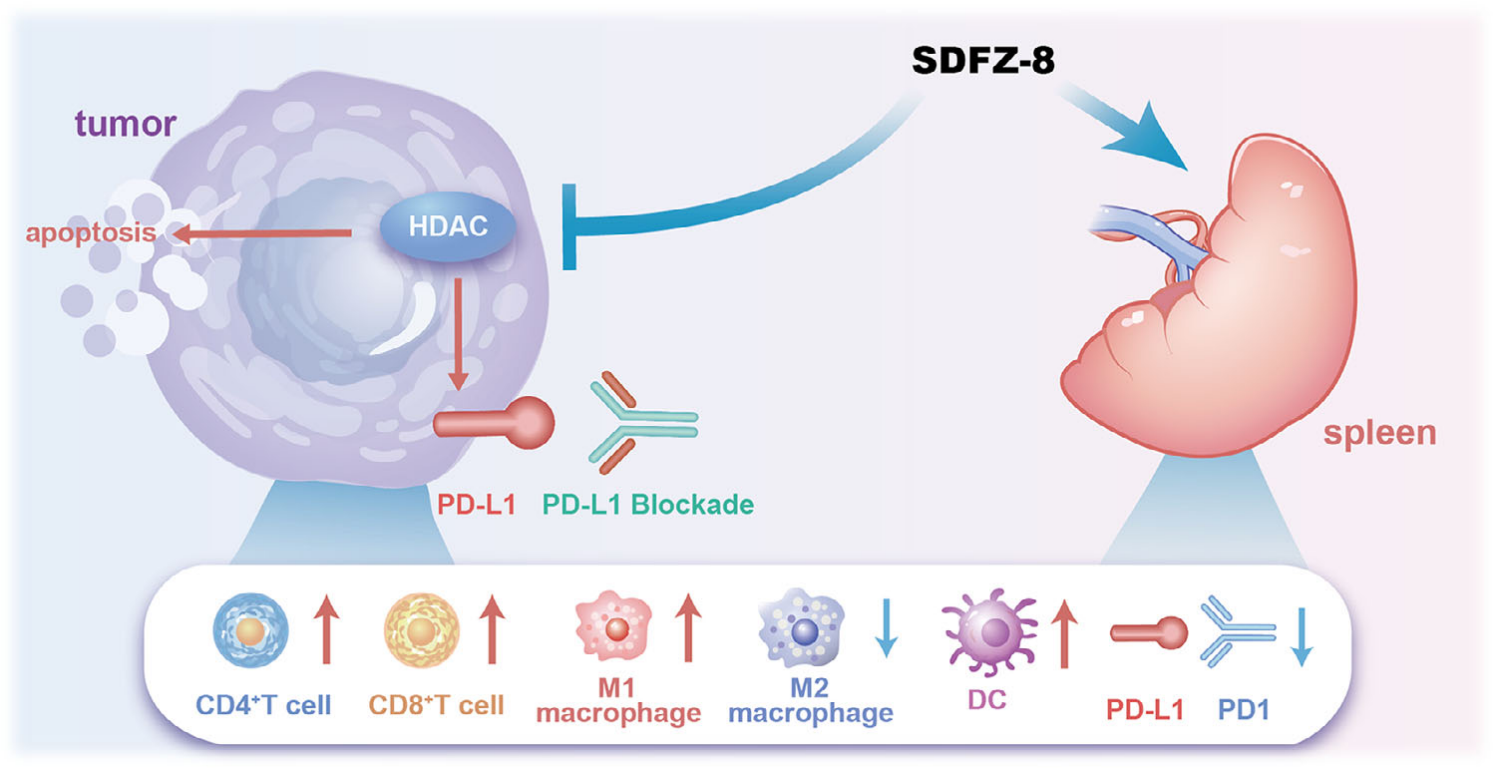
Proposed mechanism of action of **SDFZ‐8** in stimulating antitumor immunity.

In comparison with nonselective HDAC inhibitors, our newly designed HDAC1/2/6 inhibitor, **SDFZ‐8**, not only demonstrated more potent T cell activation and improved antigen presenting ability but also contributed to alleviating the immunosuppressive tumor environment by promoting macrophage polarization and negatively regulating PD‐1 expression (Figure 7). Interestingly, **SDFZ‐8** has demonstrated a notable ability to elevate PD‐L1 expression in both in vitro and in vivo studies, which is closely related to its potent inhibitory effect on HDAC1. The upregulation of PD‐L1 levels facilitates the antitumor immune therapeutic activity of PD‐L1 blockade and we observed increased antitumor efficacy of **SDFZ‐8** when used in combination with PD‐L1 blockade.

The epigenetic regulation plays a pivotal role in modulating the tumor immune microenvironment. Prior studies have indicated that HDAC inhibitors exhibit immunomodulatory effects; however, the specific molecular mechanisms remain unclear [39, 40, 41, 42, 43, 44, 45]. Emerging evidence suggests broader HDAC‐PD‐L1 regulatory networks. HDAC3 enhances PD‐L1 expression via STAT3/NF‐κB signaling [46], while HDAC6 promotes PD‐L1 transcription through STAT3 phosphorylation [47]. In hepatocellular carcinoma, HDAC9 correlates with PD‐L1 overexpression though its mechanism remains unclear [48]. Recent research has elucidated that HDAC2, a member of the class I HDAC family, can impact tumor immune evasion by catalyzing the acetylation of PD‐L1, thus influencing its nuclear translocation [17]. HDAC5, a member of class II HDAC family, has been found to regulate PD‐L1 expression by stimulating p65 deacetylation in pancreatic cancer [49]. Our discovery that HDAC1 inhibition upregulates PD‐L1 adds another layer to this complex regulation, suggesting isoform‐specific targeting may differentially modulate immune checkpoint expression. Also, the newly designed HDAC1/2/6 inhibitor **SDFZ‐8** not only effectively upregulates PD‐L1 expression in tumor cells but also modulates lymphocyte expression of PD‐1 and PD‐L1, thereby alleviating the suppressive immune microenvironment and enhancing the therapeutic efficacy of anti‐PD‐L1 blockade.

This study has limitations that also present opportunities for future research. The broader inhibitory profile of **SDFZ‐8**, while central to its efficacy, precludes attribution of its effects to a single HDAC isoform. Consequently, the relative contribution of HDAC1/2 versus HDAC6 inhibition to the overall antitumor and immune‐modulating effects warrants further investigation. Additionally, the exact immune‐mediated mechanisms of action require deeper validation in immunocompetent settings. Despite these open questions, our findings robustly demonstrate that simultaneous targeting of these HDACs with **SDFZ‐8** yields potent antitumor activity and underscore the therapeutic promise of this strategy in oncology.

## 4 Experimental

### 4.1 HDACs Enzymatic Assays

HDACs enzymatic assays were conducted according to our previous work [50]. In detail, the HeLa nuclear extract, HDAC1 (BPS BIOSCIENCE INC, 50051), HDAC2 (BPS BIOSCIENCE Inc; 50052), HDAC3/NCOR1 complex (BPS BIOSCIENCE Inc; 50003), HDAC4 (BPS BIOSCIENCE Inc; 50004), HDAC5 (BPS BIOSCIENCE Inc; 50005), HDAC6 (BPS BIOSCIENCE Inc; 50006), HDAC7 (BPS BIOSCIENCE Inc; 50007), HDAC8 (BPS BIOSCIENCE Inc; 50008), HDAC9 (BPS BIOSCIENCE Inc; 50009), HDAC10 (BPS BIOSCIENCE Inc; 50010), or HDAC11 (BPS BIOSCIENCE Inc; 50011) was diluted to the proper concentrations according to the relative enzymatic activity and the linear range of the plate reader and mixed with different concentrations of drugs. Substrates (Boc‐Lys(acetyl)‐AMC for HeLa nuclear extract, Ac‐Leu‐Gly‐Lys (Tfa)‐AMC for HDAC4, HDAC5, HDAC6, HDAC7, HDAC9, HDAC10, and HDAC11, Boc‐Lys(triflouroacetyl)‐AMC for HDAC8 and Ac‐Leu‐Gly‐Lys(acetyl)‐AMC for HDAC1, HDAC2, HDAC3) were added to the mixture. The blank group was set to no enzyme or drug but contains substrates and the control group was set to no drug but contains substrates and enzymes to each 96‐well plate. After 30 min of incubation at 37°C, the mixture was terminated with 1 mg/mL trypsin and 3 µM TSA. After another 30 min, the fluorescence intensity was measured using a Varioskan plate reader at 360/460 nm. The inhibition rate was calculated by the formula: inhibition rate (%) = (fluoresce of control group − fluoresce of experiment group)/(fluoresce of control group − fluoresce of blank group) × 100%. The inhibition curves and IC_50_ values were simulated and calculated using the log(inhibitor) versus response (three parameters) mode in GraphPad Prism software.

### 4.2 Cell Culture and CCK‐8 Assay

Cell lines purchased from the Cell Bank of Type Culture Collection of the Chinese Academy of Sciences (Shanghai, China) were passaged after resuscitation. All cells in this work were cultured under 37°C and 5% CO_2_ circumstance with RPMI‐1640 medium with 10% fetal bovine serum and 1% penicillin‐streptomycin (all purchased from Biological Industries Inc.). The third to tenth passages of cells were used for experiments. For the CCK‐8 assay, cells (4000 cells per well for solid tumors and 1 × 10^5^ cells per well for hematological tumors) were seeded in a 96‐well plate and incubated with different drugs for 48 h. After incubation, 20 µL CCK‐8 solution (MeilunBio) was added. Two hours later, absorption was measured by a Varioskan plate at 450 nm. Inhibition curves and IC_50_ values were simulated and calculated using the log(inhibitor) versus response (three parameters) mode via GraphPad Prism software.

### 4.3 Colony Formation Assay

The colony formation assay is performed by seeding 1000 MDA‐MB‐231 cells per well into 6‐well plates and allowing them to adhere overnight. After treatment with **SDFZ‐8** or **SAHA** for 48 h, cells are cultured for 14 days​​ to form colonies. Once colonies are visible (≥50 cells per colony), the medium is removed, and cells are gently washed with PBS, fixed with ​​4% paraformaldehyde for 15 min, and stained with ​​Giemsa stain​​ solution for 30 min. Excess stain is rinsed with water, and plates are air‐dried. Colonies are then imaged.

### 4.4 EdU Assay

The EdU assay is performed by BeyoClick EdU kit. MDA‐MB‐231 cells were cultured in a 6‐well plate overnight. After treatment with **SDFZ‐8** or **SAHA** for 24 h, cells are labeled with a ​​2×EdU working solution and incubated for ​​2 h. Following labeling, cells are fixed with ​​4% PFA for 15 min, washed three times with wash buffer, and permeabilized using ​​0.3% Triton X‐100 for 15 min. After another wash, endogenous peroxidases are blocked for 20 min. For detection, a ​​click reaction mix​​ (containing Biotin Azide, CuSO_4_, and click additive) is added and incubated for 30 min. After washing, ​​streptavidin–HRP​​ is applied and reacted for 30 min, followed by ​​5 min DAB substrate​ incubation​. Finally, cells are washed, optionally counterstained, and imaged under a microscope.

### 4.5 TUNEL Assay

The TUNEL assay was performed with the one‐step TUNEL apoptosis kit (MeilunBio) according to the manual from the manufacturer [50, 51]. Briefly, cells (5 × 10^5^ cells per plate) were seeded in confocal dishes and incubated with drugs for 24 h. The medium was removed, and cells were fixed with 4% polyoxymethylene solution for 30 min. Subsequently, cells were treated with 20 µg/mL protease K for 5 min and TUNEL working solution for 1 h at 37°C. Eventually, cells were captured with a Leica TCS SP8 super‐resolution laser scanning confocal microscope.

### 4.6 Annexin V‐FITC/PI Dual Staining Assay

The dual staining apoptosis assay was performed with the Annexin V‐FITC/PI apoptosis kit, followed by the manufacturer's instructions [50, 51, 52]. Generally, cells (3 × 10^5^ cells per well) were seeded in six‐well plates and treated with drugs for 24 h. After incubation, cells were harvested and dyed with Annexin V‐FITC and PI solutions for 15 min. Flow cytometry was performed to analyze cell apoptosis with a Beckman Coulter Gallios Flow Cytometer.

### 4.7 GreenNuc Assay for the Detection of Caspase‐3 Activation

Caspase‐3 activation was detected with the GreenNuc caspase‐3 assay kit for live cells (Beyotime Inc). Cells (10^5^ cells per well) were seeded in black 96‐well plates and treated with different concentrations of compounds. After 24 h, the medium was discarded, and cells were treated with GreenNuc caspase‐3 substrate at room temperature for 30 min. Fluorescence intensity was measured using a Varioskan plate reader at 485/515 nm, and the final activation folds were calculated.

### 4.8 Molecular Docking and Pocket Calculations

*LigPrep* in the Schrodinger suite was used to process target compounds. OPLS_2005 force field was selected, and the pH was set to 7.0 ± 2.0. Other parameters were kept as default. Subsequently, human HDAC1 (PDB ID: 5ICN) or HDAC2 (PDB ID: 4LXZ) were obtained from RCSB PDB (https://www.rcsb.org), and the protein preparation wizard was used to perform pretreatments such as removing waters, hydrogenating, charging, and adjusting the protonation state of histidine residues near zinc ions [53]. Next, the ligand in the processed protein was chosen to generate a docking grid with 20 Å, and the remaining parameters were kept as default. The Glide module in the Schrodinger suite was used to dock the processed ligands and the docking grid. The docking accuracy was XP mode, and the remaining options remain the default [54]. Fragment‐centric topographical mapping (FCTM) of protein‐ligand binding area was performed using *AlphaSpace* [28] and the figures were illustrated by UCSF Chimera [55].

### 4.9 Immunoblotting Assay

Anti‐H3 (#13998), anticleaved Caspase‐3 (#9661) and anti‐PARP (#9542) antibody was purchased from Cell Signal Technology (CST). Anti‐Caspase‐3 (T40044) was brought from Abmart. Antitubulin antibody (ab179484) was obtained from Abcam PLC. Anti‐Bcl‐2 (F0125) and anti‐Bax (F0037) antibody were purchased from Selleck Chemicals. Anti‐GAPDH antibody (AF1186) was obtained from Beyotime Inc. The protocol of Western blotting was the same as our previous works [50]. In brief, 2.5 × 10^5^ MDA‐MB‐231 cells were treated with different drugs or DMSO (equal volume to experimental groups) for 24 h and lysed with modified RIPA buffer. The cell lysates were added to loading buffer (Beyotime #P0015L) and heated in boiling water for 5 mins and then, together with biomarker (Epizymebiotech; 15–250 kDa), were electrophoresed in SDS‐PAGE gels under 80 V voltage until the biomarker reached the end of gels. Next, SDS‐PAGE gels and PVDF membrane were clipped together to perform transmembrane procedure under 350 mA currency for 1 h. Then after incubation with 5% skim milk, TBST, primary antibody and secondary antibody, the PVDF membrane was treated with ECL chemiluminescence solution (Boster Biological Technology #AR1174) and imaged by the imager (Imager 680 Amersham Imager).

### 4.10 Immunofluorescence Assay

Cells (10^5^ cells per plate) were seeded in confocal dishes and incubated with drugs or DMSO (equal volume to experimental groups) for 24 h. The medium was removed, and cells were fixed with 4% polyoxymethylene solution for 30 min. The cells were treated with 0.2% Triton X‐100 for 10 min and 1% BSA for 1 h at room temperature. After that, cells were washed twice with TBST and incubated with anti‐H3 antibody (Cell Signal Technology; #13998) at 4°C overnight. When incubation finished, cells were washed five times with TBST and Cy3‐labeled goat anti‐rabbit IgG (Beyotime; #A0516) and DAPI solution (Solarbio) were added. After 1 h incubation and washing five times with TBST, cells were captured with Leica TCS SP8 super‐resolution laser scanning confocal microscope. The colocalization of ac‐H3 and DAPI was analyzed with ImageJ.

### 4.11 shRNA Transfection and Flow Cytometry

MDA‐MB‐231 cells in logarithmic growth phase were inoculated into six‐well plates with a cell density of 3 × 10^4^ cells per well. After incubated at 37°C for 24 h, the original medium was abandoned and 900 µL complete medium was added, in which HitransG P infection solution was added. The virus dosage was calculated according to the formula and added to the cell. Virus volume = cell number × MOI value/virus titer. After incubated in the incubator at 37°C for 16 h, replace fresh complete medium. After that, the medium changed every 24 h. 48 h after infection, the cells were treated with or without compound at a concentration of 1 µM. After 48 h of culture, the fluorescence expression was observed under microscope, and the silencing effect of shHDAC1 was detected by Western Blot. The antibodies HDAC1 (Abmart; #T55154) and GAPDH (Beyotime; #AF2819) were used. The HDAC1‐siRNA lentivirus (shHDAC1) and negative control lentivirus (shCtrl) were obtained from Shanghai GeneChem Co., Ltd. (Shanghai, China).

Next, cells were digested into single‐cell suspension with trypsin, collected in a flow tube and washed with precooled 1 × PBS. Mouse serum was added to cells for 30 min at room temperature to block cells. Then the cells were incubated with anti‐human‐PD‐L1 (BioLegend; #329732) at 4°C for 1 h, and flow cytometry was performed with FACS Celesta (BD Biosciences, USA). Use FlowJo software to analyze the data.

### 4.12 In Vitro Metabolic Assay

Compounds were prepared in a working solution of 2 mg/mL with methanol. Mouse liver microsomes (4 mg/mL; Research Institute for Liver Disease (Shanghai) Co., Ltd) were mixed with NADPH (the final concentration is 4 mM), MgCl_2_ (the final concentration is 6 mM), and shaken at 37°C for 5 min. Then, compound solutions were added to the system and incubated for 0, 10, 20, 30, 60, 90, and 120 min at 37°C under shaking conditions. The mixture was quenched with cold acetonitrile and analyzed with a Shimadzu LC‐20AT system and a C18‐silica column (Thermo; 4.6 × 250 mm, 5 µm).

### 4.13 In Vivo Pharmacokinetic Study

The compound was prepared in a 1 mg/mL working solution using DMSO/solutol/saline. SD rats (6–8 weeks old, 180–200 g, male) were purchased from Beijing HFK Bioscience Co., Ltd. (Beijing, China) and fed in accordance with the guidelines for the care and use of laboratory animals of the ethical committee of Shandong University. The doses were 2.5 mg/kg (i.v.) and 10 mg/kg (i.g.), with three animals per treatment group. Blood samples were collected from the jugular vein at different time points using heparin sodium anticoagulation tubes. After anesthetizing rats with ether, blood samples were placed in an ice water mixture for 15 min, then centrifuged at 3000×*g* for 3 min to separate the plasma, and stored at −20°C. The LC–MS/MS was used to detect the concentration of the analyte at each time point, and the pharmacokinetic parameters were calculated using WinNolin8.2 software.

### 4.14 MDA‐MB‐231 Xenograft Model

BALB/c‐nu mice were obtained from Pengyue Co. Ltd. (Jinan, China) and were fed in laminar flow cabinets under specific pathogen‐free (SPF) conditions. With the approval of ethics committee of Shandong University, the animal experiments were conducted. To establish the MDA‐MB‐231 xenograft model, BALB/c‐nu mice (3–4 weeks old and 15–18 g body weight, male) were selected and injected subcutaneously into the armpits with 1.2 × 10^7^ MDA‐MB‐231 cells. By the time tumors reached the volume of 100 mm^3^, **SDFZ‐8** or **SAHA** treatment (compounds dissolved in normal saline containing 10% DMSO and 20% Cremophor EL, 60 mg/kg, qd, i.g.,) was started, with five animals per treatment group. Body weight and tumor size were measured every 3 days during the 22‐day treatment.

### 4.15 MC38 Syngeneic Model

C57BL/6J mice were obtained from HFK Bioscience (Beijing, China) and fed in laminar flow cabinets under SPF conditions. With the approval of the ethics committee of Shandong University, the animal experiments were conducted. To establish the MC38 syngeneic model, C57BL/6J mice (6–7 weeks old and 19–24 g body weight, male) were selected and injected subcutaneously into the armpits with 1.0 × 10^6^ MC38 cells. By the time tumors reached certain volumes, **SDFZ‐8** treatment (**SDFZ‐8** in PBS solution containing 5% DMSO, 60 mg/kg, qd, i.g.) was started, with five animals per treatment group. Body weight and tumor size were measured every day during the 14‐day treatment.

After 14 days, the tumors and spleens were split, and cells were isolated to analyze the change in cancer immunity. For TILs, the tumors were incubated with the tumor digestion fluid (collagenase and hyaluronic acid included) and then filtered with a 200‐gauge mesh and centrifuged (100×*g*, 1 min). The supernatant was centrifuged (400×*g*, 5 min) and precipitated cells were purified by 40% percoll solution followed by RBC lysis and washing. For splenocytes, the spleen was passed through a 200‐gauge mesh, and splenocytes were collected after RBC lysis and washing.

The prepared cell suspensions were incubated with rat serum at room temperature for 30 min to block the FC‐receptor and incubated with fluorescent antibodies at 4°C for 1 h. For Foxp3 staining, Foxp3/Transcription Factor Staining Buffer Set (eBioscience; #00‐5523‐00) was used. For intracellular cytokines staining, cells were pre‐stimulated with 1 µg/mL ionomycin (Beyotime; #S1672), 50 ng/mL PMA (Sigma; #P1585) and Brefeldin A (BioLegend; #420601) for 4 h. The antibodies used in this work were listed in **Table** **S3**. Flow cytometry was conducted with FACS Celesta (BD Biosciences, USA) and analyzed with FlowJo software.

### 4.16 B16F10 syngeneic Model

With the approval of the ethics committee of Shandong University, C57BL/6J mice were purchased from Charles River and fed in SPF conditions at the Model Animal Research Centre of Shandong University. To establish the B16F10 syngeneic model, cells in logarithmic growth phase were trypsinized with 0.25% trypsin, resuspended in sterile PBS, and adjusted to 1 × 10^6^ cells/100 µL. The cell suspension (100 µL) was subcutaneously injected into the left axilla. When tumors reached to certain volume, mice were randomized (*n* = 5/group) and treated with Chidamide, LBH‐589, or SDFZ‐8 by oral gavage and intraperitoneal injection of anti‐PD‐L1. Tumor growth curves were plotted over a 7‐day treatment cycle. All procedures complied with AAALAC International guidelines for humane endpoint criteria.

### 4.17 H&E Staining

The tissue of organs was fixed and embedded in paraffin and cut into 5 µm sections. Tissue sections were stained with H&E for histopathological evaluation. Briefly, the discs were immersed in two variations of xylene and a range of reduced alcohol concentrations and finally immersed in single‐distilled water. The next step was staining with Harris's hematoxylin reagent for 5 min, decolorizing with 1% Hydrochloric acid ethanol for 30 s and rinsing with tap water. The sections were then stained with eosin for 5 min and washed off with tap water. After dehydration with alcohol and xylene, the sections were sealed with neutral resin and the histopathologic change was observed with microscopy.

### 4.18 Statistical Analysis

Groups were compared using unpaired two‐tailed *t*‐tests and two‐way analysis of variance was performed when comparing multiple samples. A *p* value less than 0.05 was considered statistically significant (*compared with vehicle, *p < 0.05, **p < 0.01, ***p < 0.001, ****p < 0.0001). All statistical analysis was conducted using GraphPad Prism version 6 software (San Diego, CA).

## Author Contributions

**Yi Zhou**: writing—review and editing, methodology, investigation, data curation, and funding acquisition. **Jintong Du**: writing—review and editing, methodology, investigation, and data curation. **Xue Li**: writing—review and editing, methodology, investigation, and data curation. **Huajun Zhao**: writing—review and editing, methodology, and investigation. **Junxin Xue**: writing—review and editing and investigation. **Yuchen Liu**: writing—review and editing and investigation. **Xinying Yang**: writing—review and editing, data curation, and investigation. **Jinming Yu**: writing—review and editing and conceptualization. **Xuben Hou**: writing—original draft, conceptualization, project administration, and funding acquisition. **Hao Fang**: writing—review and editing, investigation, writing—review and editing, project administration, funding acquisition, and conceptualization. All authors have read and approved the final manuscript.

## Conflicts of Interest

The authors declare no conflicts of interest.

## Ethics Statement

All experiments were approved by the Institutional Animal Care and Use Committee of Shandong University and carried out following the Guide for the Care and Use of Laboratory Animals (Approval No. 240138). All tumor dimensions in our mouse xenograft models adhered to the specified limits.

## Supporting information



Figure S1: Inhibition curves used to determine the IC_50_ values of target compounds against HeLa unclear extract.
Figure S2: Inhibition curves used to determine the IC_50_ values of SDFZ‐8 and SAHA against different HDACs.
Figure S3: Co‐localization analysis of ac‐H3 immunofluorescence and cell nuclear in 1 µM SDFZ‐8 treated MDA‐MB‐231 cells for 24 h: (A) Immunofluorescence of the ac‐H3, (B) Imaging of nuclear target dye DAPI, (C) The distribution of fluorescence intensity on the line, (D) Dot‐plot of the two channels.
Figure S4: Inhibition curves used to determine the IC50 values of SDFZ‐8 and SAHA against different cancer cells for 48 h treatment.
Figure S5: Metabolic stability of SDFZ‐8 in mouse liver microsomes: ^a^R^2^ is the coefficient of determination of the nonlinear regression of the data: ^b^CLint (mic) is the intrinsic clearance in the unit of µL/min/mg.
Figure S6: Change of body weight of nude mice after administration of SDFZ‐8 and SAHA in the MDA‐MB‐231 xenograft model (A), MC38 syngeneic model (B) and the B16F10 melanoma model (C).
Figure S7: Hematoxylin‐eosin staining results of the lung, kidney, liver, heart, spleen and tumor of each animal group in the MC38 syngeneic model.
Figure S8: Impacts of SAHA and SDFZ‐8 on immune cells in the spleen.
Figure S9: ^1^H‐NMR spectrum of SDFZ‐8.
Figure S10: ^13^C‐NMR spectrum of SDFZ‐8.
Figure S11: HRMS spectrum of SDFZ‐8.
Table S1: Antiproliferation activity of selected compounds^a^.
Table S2: Summary of in vivo anti‐tumor activities.
Table S3: Information for the antibodies used in tumor immunity study.

## Data Availability

Data will be made available on request.
