# Supplementary material for: Preclinical Characterization of SDFZ‐8, a Highly Potent HDAC1 Inhibitor, for Cancer Immunotherapy
Source: MedComm (2020). 2025 Nov 18;6(12):e70500. doi: 10.1002/mco2.70500 (PMC12627232; doi:10.1002/mco2.70500)
Supplement: Supplementary file 1 — Figure S1: Inhibition curves used to determine the IC50 values of target compounds against HeLa unclear extract. Figure S2: Inhibition curves used to determine the IC50 values of SDFZ‐8 and SAHA against different HDACs. Figure S3: Co‐localization analysis of ac‐H3 immunofluorescence and cell nuclear in 1 µM SDFZ‐8 treated MDA‐MB‐231 cells for 24 h: (A) Immunofluorescence of the ac‐H3, (B) Imaging of nuclear target dye DAPI, (C) The distribution of fluorescence intensity on the line, (D) Dot‐plot of the two channels. Figure S4: Inhibition curves used to determine the IC50 values of SDFZ‐8 and SAHA against different cancer cells for 48 h treatment. Figure S5: Metabolic stability of SDFZ‐8 in mouse liver microsomes: aR2 is the coefficient of determination of the nonlinear regression of the data: bCLint (mic) is the intrinsic clearance in the unit of µL/min/mg. Figure S6: Change of body weight of nude mice after administration of SDFZ‐8 and SAHA in the MDA‐MB‐231 xenograft model (A), MC38 syngeneic model (B) and the B16F10 melanoma model (C). Figure S7: Hematoxylin‐eosin staining results of the lung, kidney, liver, heart, spleen and tumor of each animal group in the MC38 syngeneic model. Figure S8: Impacts of SAHA and SDFZ‐8 on immune cells in the spleen. Figure S9: 1H‐NMR spectrum of SDFZ‐8. Figure S10: 13C‐NMR spectrum of SDFZ‐8. Figure S11: HRMS spectrum of SDFZ‐8. Table S1: Antiproliferation activity of selected compoundsa. Table S2: Summary of in vivo anti‐tumor activities. Table S3: Information for the antibodies used in tumor immunity study. [file MCO2-6-e70500-s001.pdf]

Supporting Information

Preclinical characterization of SDFZ-8, a highly potent HDAC1 inhibitor, for cancer immunotherapy

Yi Zhou [1, 3,#], Jintong Du [2,#], Xue Li [1,#], Huajun Zhao [4], Junxin Xue [1], Yuchen Liu [1], Xinying Yang [1], Jinming Yu [2,*], Xuben Hou [1,*] and Hao Fang [1,*]

[1] *Department of Medicinal Chemistry, State Key Laboratory of Discovery and Utilization of Functional Components in Traditional Chinese Medicine, Shandong Key Laboratory of Druggability Optimization and Evaluation for Lead Compounds, School of Pharmaceutical Science, Cheeloo College of Medicine, Shandong University, Ji'nan, Shandong, 250012, P.R. China*

[2] *Shandong Cancer Hospital and Institute, Shandong First Medical University, Ji'nan, Shandong, 250117, P.R. China*

[3] *Department of Pharmacy, Shandong Provincial Hospital Affiliated to Shandong First Medical University, Ji'nan, Shandong, 250021, P.R. China*

[4] *Department of Immunopharmaceutical Sciences, School of Pharmaceutical Science, Cheeloo College of Medicine, Shandong University, Ji'nan, Shandong, 250012, P.R. China*

*** Corresponding authors:**

Prof. Hao Fang, Email: haofangcn@sdu.edu.cn

Prof. Xuben Hou, Email: hxb@sdu.edu.cn

Prof. Jinming Yu, Email: sdyujinming@163.com

Note

[#] *These authors contribute equally to this work*

Table of Contents

1. Supplementary Figures S1-S11

2. Supplementary Tables S1-S3

3. Chemistry

1. Supplementary Figures S1-S11

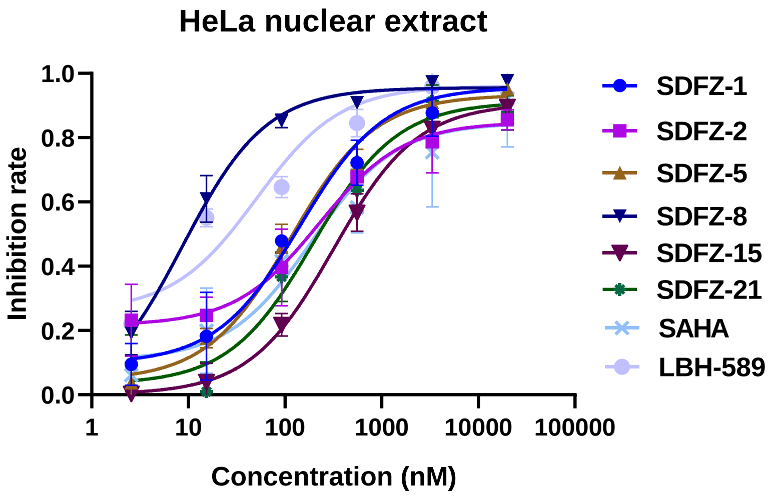

Figure S1. Inhibition curves used to determine the IC_{50} values of target compounds against HeLa unclear extract.

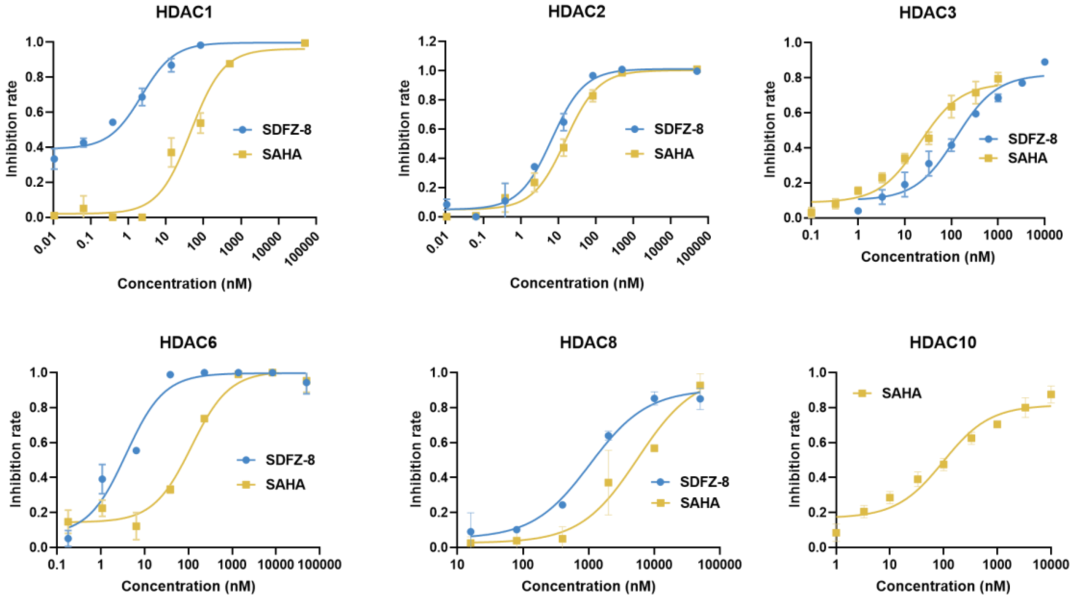

Figure S2. Inhibition curves used to determine the IC_{50} values of **SDFZ-8** and **SAHA** against different HDACs.

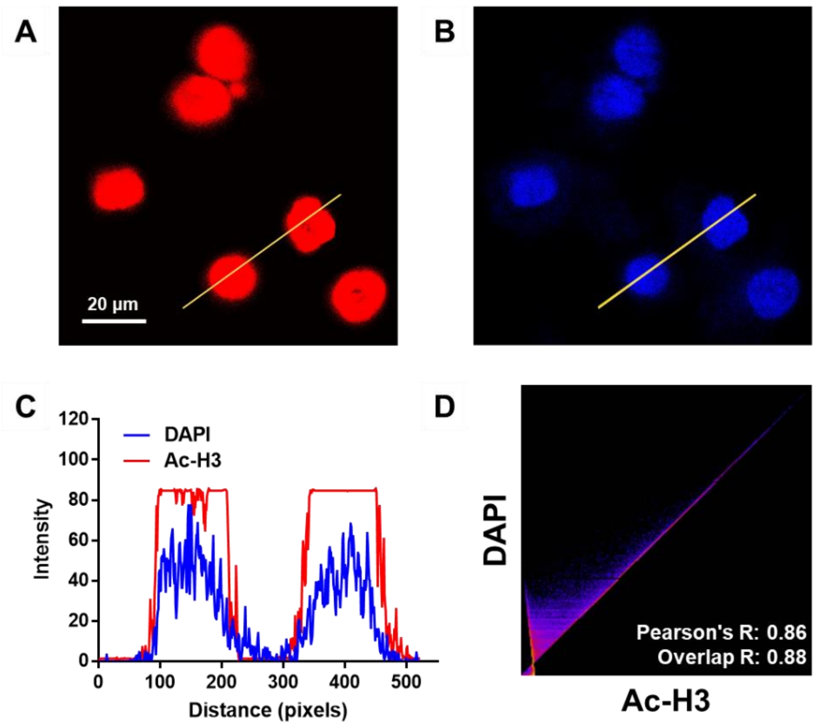

Figure S3. Co-localization analysis of ac-H3 immunofluorescence and cell nuclear in 1 μM **SDFZ-8** treated MDA-MB-231 cells for 24 h. (A) Immunofluorescence of the ac-H3, (B) Imaging of nuclear target dye DAPI, (C) The distribution of fluorescence intensity on the line, (D) Dot-plot of the two channels.

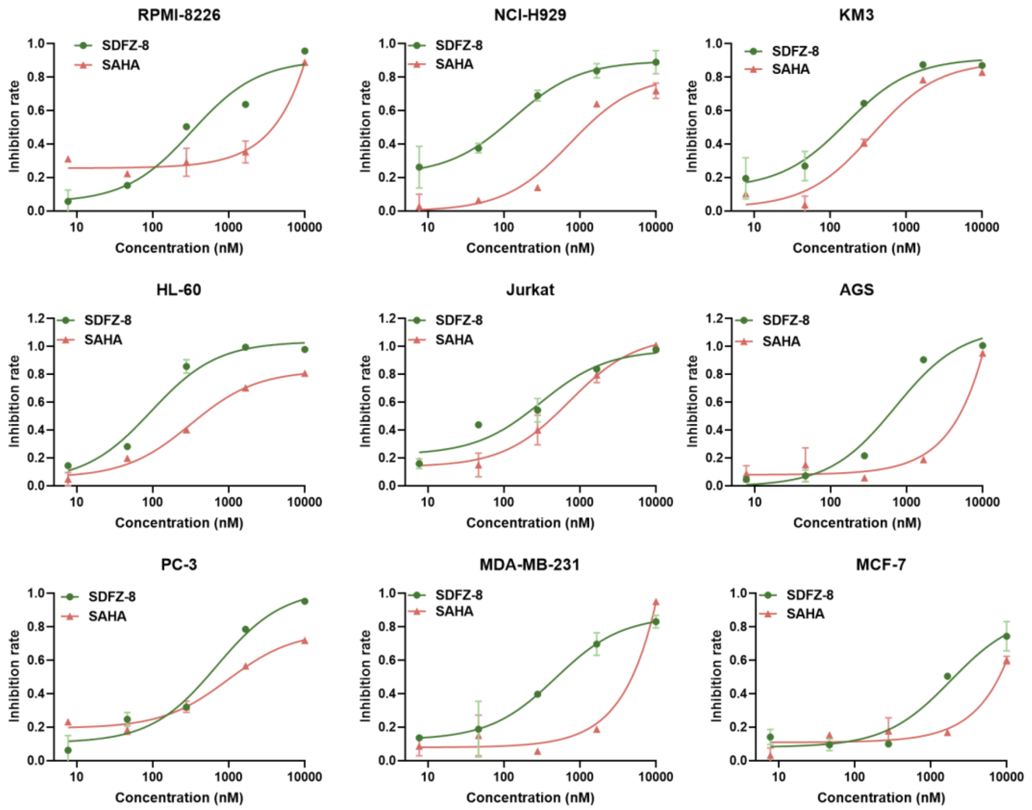

Figure S4. Inhibition curves used to determine the IC_{50} values of **SDFZ-8** and **SAHA** against

different cancer cells for 48 h treatment.

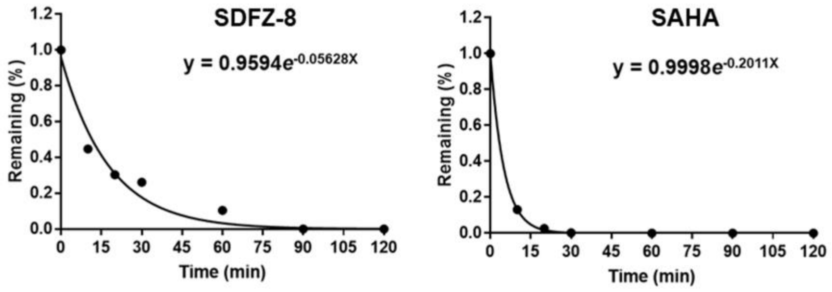

	$R^{2\,a}$	$T_{1/2}$ (min)	$CL_{int(mic)}$ (μL/min/mg) [b]
**SDFZ-8**	0.97	12.31	56.28
**SAHA**	0.99	3.45	201.1

Figure S5. Metabolic stability of **SDFZ-8** in mouse liver microsomes. [a] R^2 is the coefficient of determination of the nonlinear regression of the data. [b] $CL_{int(mic)}$ is the intrinsic clearance in the unit of μL/min/mg.

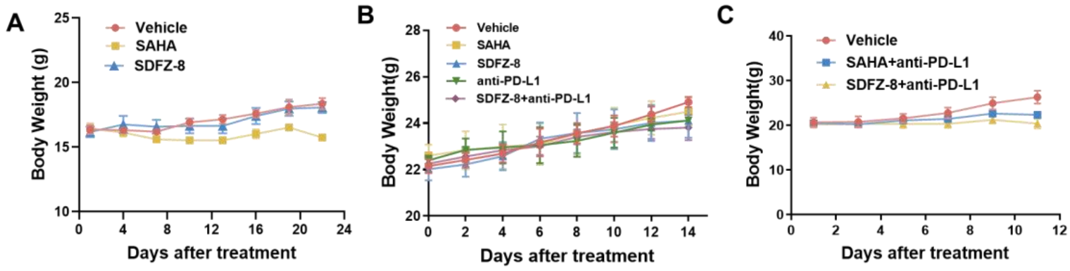

Figure S6. Change of body weight of nude mice after administration of **SDFZ-8** and **SAHA** in the MDA-MB-231 xenograft model (A), MC38 syngeneic model (B) and the B16F10 melanoma model (C).

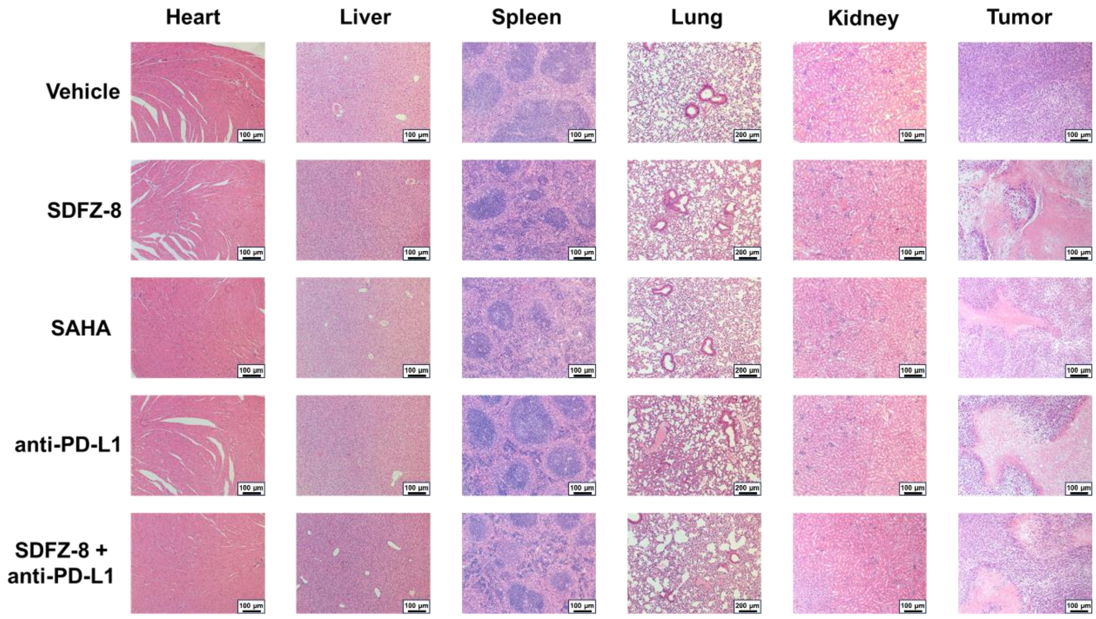

Figure S7. Hematoxylin-eosin staining results of the lung, kidney, liver, heart, spleen and tumor of

each animal group in the MC38 syngeneic model.

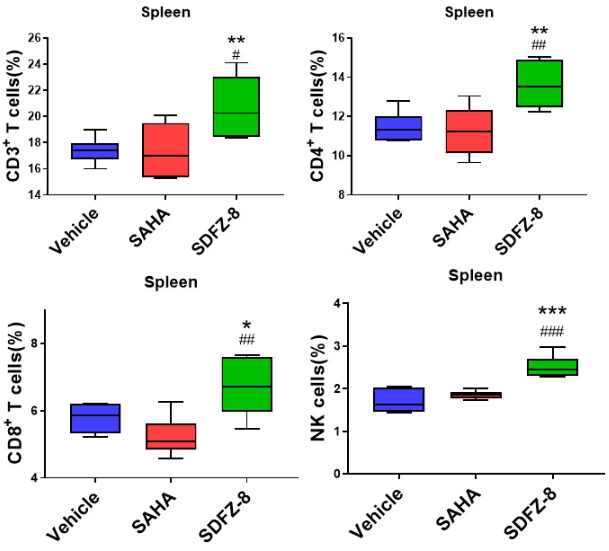

Figure S8. Impacts of **SAHA** and **SDFZ-8** on immune cells in the spleen.

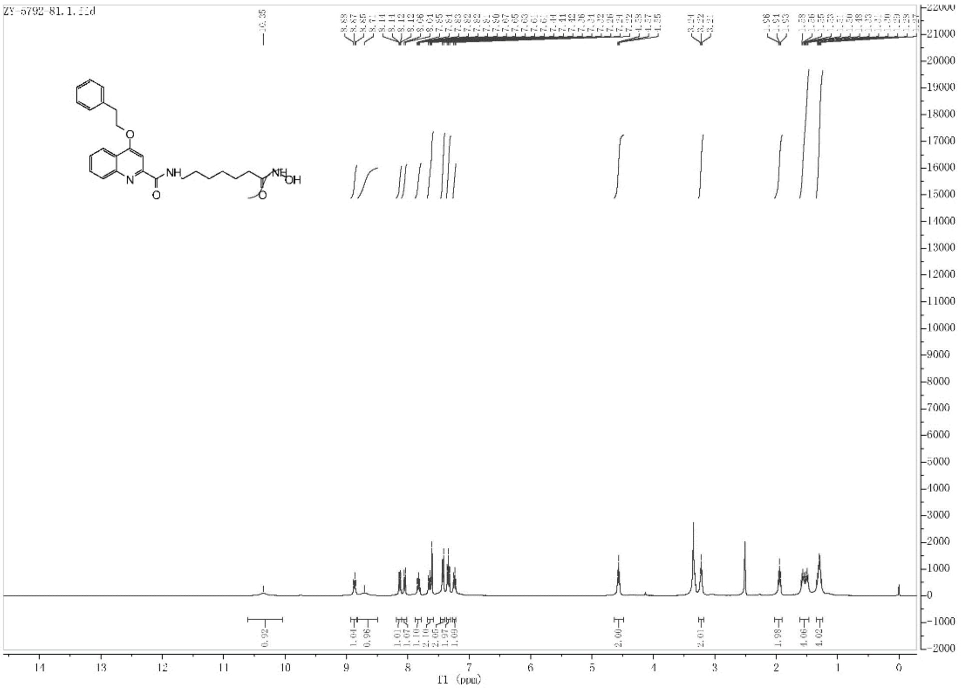

Figure S9. ^{1}H-NMR spectrum of **SDFZ-8**

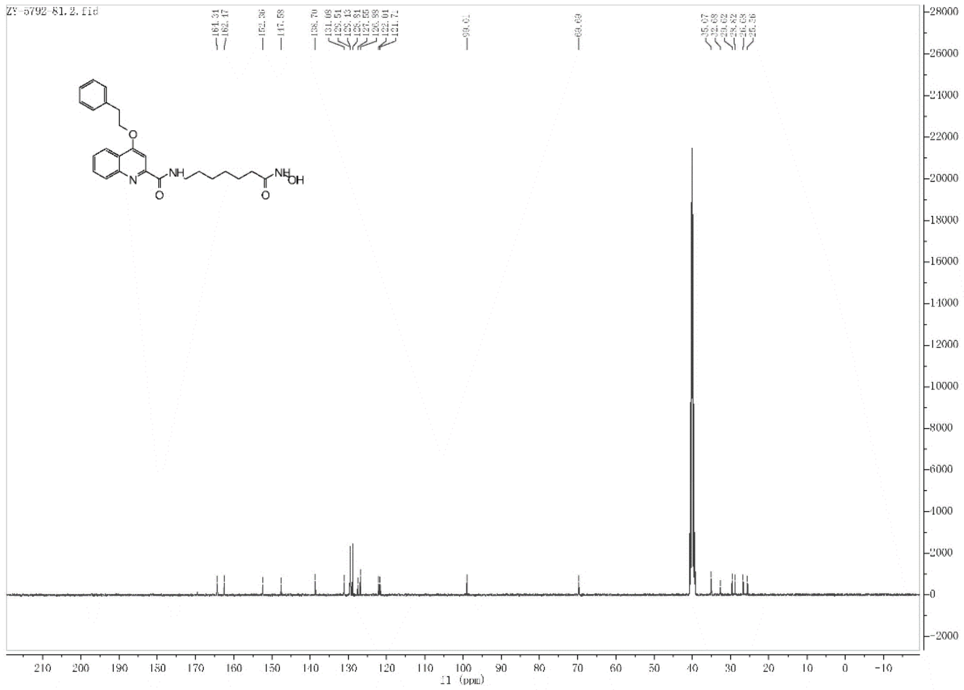

Figure S10. ^{13}C-NMR spectrum of **SDFZ-8**

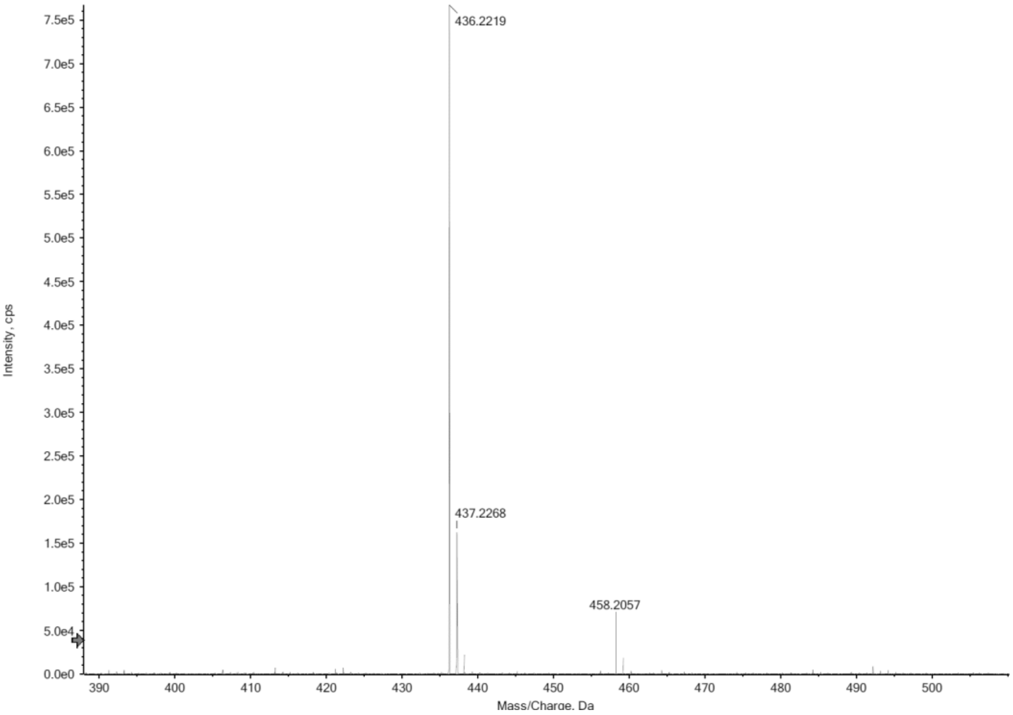

Figure S11. HRMS spectrum of **SDFZ-8**

2. Supplementary Tables S1-S3

Table S1. Antiproliferation activity of selected compounds [a]

Cancer cells	IC$_{50}$ values (μM) [a]	
	SDFZ-8	SAHA
**RPMI-8226**	0.46±0.03	2.4±0.5
**NCI-H929**	0.100±0.005	0.89±0.14
**KM3**	0.100±0.005	0.34±0.08
**HL-60**	0.094±0.004	0.292±0.005
**Jurkat**	0.089±0.001	0.55±0.13
**AGS**	0.56±0.02	2.5±0.6
**PC-3**	0.62±0.07	1.04±0.03
**MDA-MB-231**	0.47±0.08	1.4±0.3
**MCF-7**	1.96±0.06	8.4±0.7

[a] Results are expressed as the mean ± SD of three separate determinations.

Table S2. Summary of in vivo anti-tumor activities.

Group	TGI values [a]		
	MDA-MB-231 model	MC38 model	B16F10 model
**SAHA**	60%	50%	-
**LBH-589**	-	-	53%
**Chidamide**	-	-	58%
**SDFZ-8**	76%	71%	75%
**anti-PD-L1**	-	64%	62%
**LBH-589+anti-PD-L1**	-	-	77%
**Chidamide+anti-PD-L1**	-	-	75%
**SDFZ-8+anti-PD-L1**	-	86%	91%

[a] TGI value= (1-$RTV_{experimental}$/$RTV_{control}$) *100%, where RTV = relative tumor volume.

Table S3. Information for the antibodies used in tumor immunity study.

Antibody	Species	Cat	Supplier
IFN-γ	Mouse	505830	BioLegend
Fixable Viability Dye	Mouse	65-0866-14	eBioscience
PD-L1	Mouse	124321	BioLegend
CD11c	Mouse	11-0114-85	eBioscience
CD4	Mouse	11-0041-85	eBioscience
CD11b	Mouse	12-0112-83	eBioscience
TNF-α	Mouse	25-7321-82	eBioscience
iNOS	Mouse	12-5920-83	eBioscience
CD8a	Mouse	562283	BD Biosciences
F4/80	Mouse	45-4801-82	eBioscience
CD3e	Mouse	45-0031-82	eBioscience
PD-1	Mouse	135210	BioLegend
CD206	Mouse	141708	BioLegend
NK 1.1	Mouse	108724	BioLegend
MHC II	Mouse	107652	BioLegend
CD45.2	Mouse	109822	BioLegend

3. Chemistry

The synthetic routes of target compounds are illustrated in **Schemes S1-S3**. Initially, kynurenic acid methyl ester was synthesized from aniline(1). Subsequently, a series of different compounds including methyl iodide, (bromomethyl)benzene, 1-(bromomethyl)-4-(tert-butyl)benzene, (2-chloroethyl)benzene, 1-(bromomethyl)-4-fluorobenzene, 1-(bromomethyl)-4-methoxybenzene, (2-bromoethyl)benzene, 4-(bromomethyl)-1,1'-biphenyl or 1-(bromomethyl)naphthalene were reacted with **3** using the Williamson reaction to introduce 4-position substituent groups, resulting in the synthesis of intermediates **4a-4i** (2). Following demethylation, intermediates **5a-5i** were obtained. Moving on to **SDFZ-1** to **SDFZ-8**, as depicted in **Scheme S1**, different amino acid methyl esters were connected to the 2-position of quinoline via amide bonds to obtain **6a-6k**(3). The target compounds were then obtained by transforming these into hydroxamic acids(4). For **SDFZ-9** to **SDFZ-21**, in **Scheme S2**, **5a** to **5m** were reacted with intermediates **10a** or **10b**, obtained via the Heck reaction, and subsequently reacted with hydroxylamine to yield the target compounds in the form of hydroxamic acids(5).

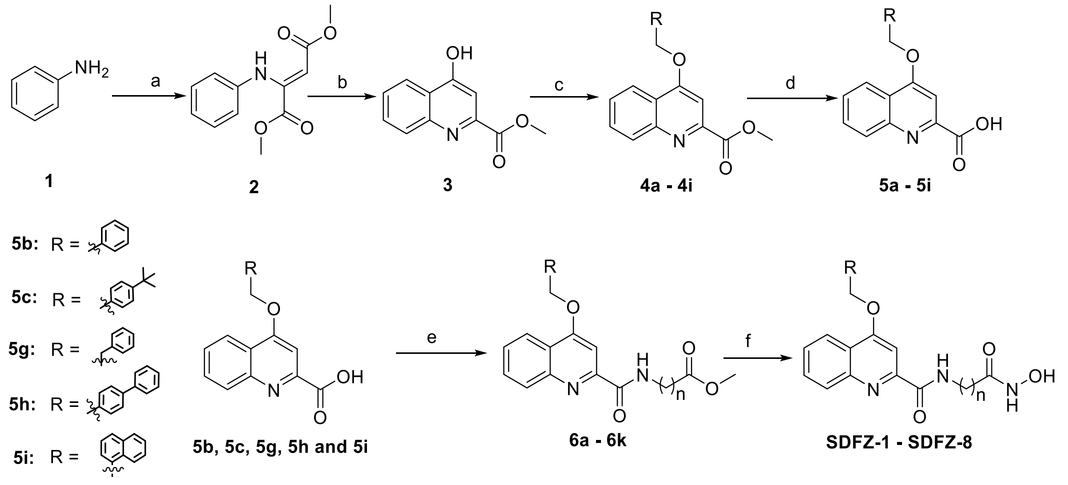

Scheme 1 Synthetic route of target compounds **SDFZ-1-SDFZ-8**. Reagents and conditions: (a) dimethyl butynedioate, methanol, 0°C to 80°C, 8 h; (b) polyphosphoric acid, 120°C, 2h; (c) methyl iodide, (bromomethyl)benzene, 1-(bromomethyl)-4-(tert-butyl)benzene, (2-chloroethyl)benzene, 1-(bromomethyl)-4-fluorobenzene, 1-(bromomethyl)-4-methoxybenzene, (2-bromoethyl)benzene, 4-(bromomethyl)-1,1'-biphenyl or 1-(bromomethyl)naphthalene, DMF (*N*,*N*-dimethylformamide), K_2CO_3, 2h; (d) LiOH, H_2O/THF (tetrahydrofuran); (e) 6-methoxy-6-oxohexan-1-amine hydrochloride or 7-methoxy-7-oxoheptan-1- amine hydrochloride, HATU (2-(7-azabenzotriazol-1-yl)-*N*,*N*,*N'*,*N'*-tetramethyluronium hexafluorophosphate), NMM (*N*-methylmorpholine), DMF, room temperature, 8 h; (f)

hydroxylamine hydrochloride, KOH, methanol, room temperature, overnight.

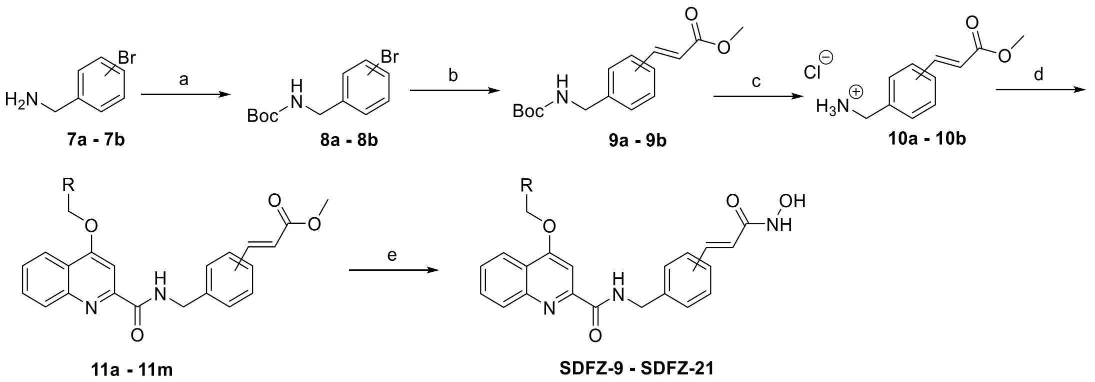

Scheme 2 The synthetic route of target compounds **SDFZ-9-SDFZ-21**. Reagents and conditions: (a) triethylamine, di-*tert*-butyl dicarbonate, 8h, room temperature; (b) methyl acrylate, triethylamine, tetrakistriphenylphosphine palladium, 130°C, 12 h; (c) saturated ethyl acetate solution of hydrogen chloride; (d) **5a-5m**, HATU, NMM, DMF, room temperature, 8h; (e) hydroxylamine hydrochloride, KOH, methanol, room temperature, overnight.

All reagents and solvents are analytical grade and commercially available. All ^{1}H-NMR and ^{13}C-NMR spectra were measured with a Bruker DRX spectrometer at 400 MHz or 800MHz and TMS as the internal standard. The HRMS spectra were determined by the SCI-EX X500e Q-TOF LC/MS system. All melting points were measured with an electrothermal melting point meter. The purity of the target compound was determined by reversed-phase high-performance liquid chromatography (RP-HPLC) with Shimadzu LC-20AT system and a C18 silica gel column (Thermo, 4.6 mm×250 mm, 5 μm). The mobile phase was acetonitrile/water (60:40), the flow rate was 1 mL/min, and the measurement reached a minimum of 95% purity.

3.1 Dimethyl 2-(phenylamino)fumarate (2)

Aniline (20.00 g, 0.22 mol) was dissolved in 40 mL methanol, and dimethyl butynedioate (30.54 g, 0.22 mol) was added dropwise at 0°C. Subsequently, the reaction solution was heated to 80 °C and refluxed for 8 hours. The solvent was vapored to obtain a yellow oily product. After sampled with silica gel, it was purified by column chromatography (petroleum ether: ethyl acetate = 99:1), and finally, a pure light yellow oily intermediate **2** (6) (35.36 g) was obtained with a yield of 70%. ^{1}H NMR (400 MHz, DMSO-d_6) δ 9.66 (s, 1H), 7.31 (t, J = 7.1 Hz, 2H), 7.08 (t, J = 7.1 Hz, 1H), 6.96 (d, J = 7.6 Hz, 2H), 5.32 (s, 1H), 3.67 (s, 6H).

3.2 Methyl 4-hydroxyquinoline-2-carboxylate (3)

Mixture intermediate **2** (30.00 g, 0.12 mol) and polyphosphoric acid (100.00 g) were stirred by a mechanical agitator and slowly heat to 120 °C. After the mixture gradually turned to red, and the temperature was kept for 2 h. Subsequently, 10% potassium carbonate solution was slowly added to the reaction solution until all the viscous liquid dissolves and no more bubbles are generated. The suspension was filtered and the filter cake was washed with a small amount of water and dried to obtain intermediate **3** (19.40 g). Brown solid, yield: 75%, melting point: 224 - 226 °C. ^{1}H NMR (400 MHz, DMSO-d$_6$) δ 12.09 (s, 1H), 8.08 (d, *J* = 8.0 Hz, 1H), 7.95 (d, *J* = 8.4 Hz, 1H), 7.72 (t, *J* = 7.6 Hz, 1H), 7.38 (t, *J* = 7.5 Hz, 1H), 6.63 (s, 1H), 3.97 (s, 3H).

3.3 Methyl 4-methoxyquinoline-2-carboxylate (4a)

Intermediate **3** (2.00 g, 10.00 mmol), methyl iodide (1.68 g, 11.00 mmol) and potassium carbonate (4.08 g, 30 mmol) were dissolved in 50 mL DMF and heated to 70 °C for 8 h. After naturally cooling to room temperature, the reaction solution was quenched with water. The deposit was filtered, washed with a small amount of water and dried to obtain **4a** (7) (1.57 g). Brown solid, yield: 74%, melting point: 224 - 226 °C. ^{1}H NMR (400 MHz, DMSO-*d*$_6$) δ 8.23 - 8.17 (m, 1H), 8.10 (d, *J* = 8.4 Hz, 1H), 7.86 (ddd, *J* = 8.4, 6.8, 1.5 Hz, 1H), 7.72 - 7.68 (m, 1H), 7.56 (d, *J* = 2.1 Hz, 1H), 4.13 (s, 3H), 3.96 (s, 3H).

3.4 Methyl 4-(benzyloxy)quinoline-2-carboxylate (4b)

Intermediate **3** (2.00 g, 10.00 mmol), (bromomethyl)benzene (1.12 g, 11.00 mmol) and potassium carbonate (4.08 g, 30.00 mmol) were dissolved in 50 mL DMF and heated to 70 °C for 8 h. After naturally cooling to room temperature, the reaction solution was quenched with water and extracted with ethyl acetate (3×20 mL). The combined organic layer was washed with 5% citric acid, saturated $NaHCO_3$ solution and saturated brine. After drying with anhydrous $MgSO_4$, filtered and evaporated to dryness to give **4b** (1.97 g) as brown oil, yield: 68%. ^{1}H NMR (400 MHz, DMSO-*d*$_6$) δ 8.24 (d, *J* = 8.4 Hz, 1H), 8.11 (d, *J* = 8.5 Hz, 1H), 7.87 (t, *J* = 7.7 Hz, 1H), 7.74 - 7.68 (m, 2H), 7.59 (d, *J* = 7.6 Hz, 2H), 7.48 - 7.44 (m, 2H), 5.51 (s, 2H), 3.96 (s, 3H).

3.5 Methyl 4-((4-(*tert*-butyl)benzyl)oxy)quinoline-2-carboxylate (4c)

Intermediate **3** (2.00 g, 0.10 mol), 1-(bromomethyl)-4-(tert-butyl)benzene (2.46 g, 11 mmol) and potassium carbonate (4.08 g, 30 mmol) were treated according to the preparation procedure of **4b**. **4c** (2.34 g) was obtained as black oil, yield: 65%. ^{1}H NMR (400 MHz, DMSO-*d*$_6$) δ 8.22 (d, *J* = 8.4 Hz, 1H), 8.11 (d, *J* = 8.6 Hz, 2H), 7.86 (t, *J* =

7.6 Hz, 1H), 7.71 (t, J = 4.6 Hz, 2H), 7.49 (d, J = 1.8 Hz, 3H), 5.46 (s, 2H), 3.96 (s, 3H), 1.31 (s, 9H).

3.6 Methyl 4-((4-chlorobenzyl)oxy)quinoline-2-carboxylate (4d)

Intermediate **3** (2.00 g, 10.00 mmol), (2-chloroethyl)benzene (1.54 g, 11.00 mmol) and potassium carbonate (4.08 g, 30 mmol) were treated according to the preparation procedure of **4a**. **4d** (2.34 g) was obtained as white solid, yield: 68%, melting point: 220 - 222 °C. ^{1}H NMR (400 MHz, DMSO-d_6) δ 8.29 (d, J = 8.4 Hz, 1H), 8.23 (d, J = 8.6 Hz, 1H), 7.96 (t, J = 7.8 Hz, 1H), 7.84 - 7.73 (m, 2H), 7.72 - 7.61 (m, 2H), 7.54 (s, 2H), 5.59 (s, 2H), 3.96 (s, 3H).

3.7 Methyl 4-((4-fluorobenzyl)oxy)quinoline-2-carboxylate (4e)

Intermediate **3** (2.00 g, 10.00 mmol), 1-(bromomethyl)-4-fluorobenzene (2.05 g, 11.00 mmol) and potassium carbonate (4.08 g, 30.00 mmol) were treated according to the preparation procedure of **4b**. **4e** (3.0 g) was obtained as brown oil, yield: 68%. ^{1}H NMR (400 MHz, DMSO-d_6) δ 8.16 (dd, J = 8.4, 1.4 Hz, 1H), 7.87 (d, J = 8.4 Hz, 1H), 7.80 - 7.71 (m, 2H), 7.70 - 7.62 (m, 2H), 7.57 (ddd, J = 8.2, 6.8, 1.2 Hz, 1H), 7.37 - 7.25 (m, 2H), 5.46 (s, 2H), 3.96 (s, 3H).

3.8 Methyl 4-((4-methoxybenzyl)oxy)quinoline-2-carboxylate (4f)

Intermediate **3** (2.00 g, 10.00 mmol), 1-(bromomethyl)-4-methoxybenzene (2.18 g, 11.00 mmol) and potassium carbonate (4.08 g, 30 mmol) were treated according to the preparation procedure of **4a**. **4f** (2.39 g) was obtained as white solid, yield: 75%, melting point: 190 - 192 °C. ^{1}H NMR (400 MHz, DMSO-d_6) δ 8.19 (d, J = 8.3 Hz, 1H), 8.11 - 8.05 (m, 2H), 7.66 (dd, J = 13.8, 6.7 Hz, 2H), 7.50 (s, 2H), 7.00 (s, 2H), 5.29 (s, 2H), 3.78 (d, J = 2.2 Hz, 6H).

3.9 Methyl 4-phenethoxyquinoline-2-carboxylate (4g)

Intermediate **3** (2.00 g, 10.00 mmol), (2-bromoethyl)benzene (2.00 g, 11 mmol) and potassium carbonate (4.08 g, 30.00 mmol) were treated according to the preparation procedure of **4b**. **4g** (2.24 g) was obtained as colorless oil, yield: 74%. ^{1}H NMR (400 MHz, DMSO-d_6) δ 8.14 (d, J = 8.4 Hz, 1H), 8.08 (d, J = 8.5 Hz, 1H), 7.84 (t, J = 7.7 Hz, 1H), 7.69 (t, J = 7.7 Hz, 1H), 7.55 (d, J = 1.9 Hz, 1H), 7.43 (d, J = 7.5 Hz, 2H), 7.38 - 7.31 (m, 2H), 7.27 - 7.21 (m, 1H), 4.56 (t, J = 6.5 Hz, 2H), 3.94 (s, 3H), 3.28 - 3.19 (m, 2H).

3.10 Methyl 4-([1,1'-biphenyl]-4-ylmethoxy)quinoline-2-carboxylate (4h)

Intermediate **3** (2.00 g, 10.00 mmol), 4-(bromomethyl)-1,1'-biphenyl (2.68 g, 11 mmol) and potassium carbonate (4.08 g, 30 mmol) were treated according to the

preparation procedure of **4a**. **4h** (2.10 g) was obtained as white solid, yield: 58%, melting point: >300 °C. ^{1}H NMR (400 MHz, DMSO-d_6) δ 8.27 (dd, *J* = 8.4, 1.4 Hz, 1H), 8.12 (d, *J* = 8.5 Hz, 1H), 7.88 (ddd, *J* = 8.4, 6.8, 1.5 Hz, 2H), 7.75 - 7.68 (m, 7H), 7.51 - 7.47 (m, 2H), 7.39 (dd, *J* = 5.9, 3.8 Hz, 1H), 5.57 (d, *J* = 7.9 Hz, 2H), 3.96 (s, 3H).

3.11 Methyl 4-(naphthalen-1-ylmethoxy)quinoline-2-carboxylate (4i)

Intermediate **3** (2.00 g, 10.00 mmol), 1-(bromomethyl)naphthalene (2.39 g, 11 mmol) and potassium carbonate (4.08 g, 30.00 mmol) were treated according to the preparation procedure of **4b**. **4i** (2.03 g) was obtained as yellow oil, yield: 60%. ^{1}H NMR (400 MHz, DMSO-d_6) δ 8.24 - 8.19 (m, 1H), 8.12 (dd, *J* = 8.4, 3.3 Hz, 2H), 8.02 (t, *J* = 7.1 Hz, 2H), 7.91 (s, 1H), 7.84 (t, *J* = 7.6 Hz, 2H), 7.63 (dd, *J* = 24.0, 7.7 Hz, 4H), 5.97 (s, 2H), 3.98 (d, *J* = 1.6 Hz, 3H).

3.12 4-methoxyquinoline-2-carboxylic acid (5a)

Intermediate **4a** (1.50 g, 6.91 mmol) and lithium hydroxide (0.83 g, 34.53 mmol) were dissolved in 80 mL of tetrahydrofuran/water (2/1), and the mixture was stirred at room temperature for 12 h. After the reaction, the tetrahydrofuran was vapored, and the residue was adjusted to neutral with used 2 M hydrochloric acid. The precipitate was separated by filtration, and the filter cake was washed with a small amount of water to obtain the product **5a (8)** (1.19 g). White solid, yield: 85%, melting point: >300 °C. ^{1}H NMR (400 MHz, DMSO-d_6) δ 8.14 (dd, *J* = 8.4, 1.4 Hz, 1H), 7.91 (d, *J* = 8.4 Hz, 1H), 7.76 (ddd, *J* = 8.5, 6.8, 1.4 Hz, 1H), 7.66 (s, 1H), 7.58 (ddd, *J* = 8.1, 6.8, 1.2 Hz, 1H), 4.11 (s, 3H).

3.13 4-(benzyloxy)quinoline-2-carboxylic acid (5b)

Intermediate **4b** (2.03 g, 6.91 mmol) and lithium hydroxide (0.83 g, 34.53 mmol) were treated according to the preparation procedure of **5a**. **5b** (9)(1.45 g) was obtained as white solid, yield: 75%, melting point: 272 - 274 °C. ^{1}H NMR (400 MHz, DMSO-d_6) δ 8.26 (d, *J* = 8.4 Hz, 1H), 8.15 (d, *J* = 8.5 Hz, 1H), 7.89 (t, *J* = 7.7 Hz, 1H), 7.81 - 7.67 (m, 2H), 7.60 (d, *J* = 7.5 Hz, 2H), 7.43 (dt, *J* = 26.5, 7.4 Hz, 3H), 5.53 (s, 2H).

3.14 4-((4-(tert-butyl)benzyl)oxy)quinoline-2-carboxylic acid (5c)

Intermediate **4c** (2.41 g, 6.91 mmol) and lithium hydroxide (0.83 g, 34.53 mmol) were treated according to the preparation procedure of **5a**. **5c** (1.40 g) was obtained as brown solid, yield: 64%, melting point: 286 - 288 °C. ^{1}H NMR (400 MHz, DMSO-d_6) δ 8.26 (d, *J* = 8.4 Hz, 1H), 8.19 (d, *J* = 8.5 Hz, 1H), 7.92 (t, *J* = 7.7 Hz, 1H), 7.75 (d, *J* = 7.9 Hz, 2H), 7.50 (q, *J* = 8.1 Hz, 4H), 5.52 (s, 2H), 1.31 (s, 9H).

3.15 4-((4-chlorobenzyl)oxy)quinoline-2-carboxylic acid (5d)

Intermediate **4d** (2.26 g, 6.91 mmol) and lithium hydroxide (0.83 g, 34.53 mmol) were treated according to the preparation procedure of **5a**. **5d** (1.58 g) was obtained as white solid, yield: 70%, melting point: 254 - 256 °C. ^{1}H NMR (400 MHz, DMSO-d_6) δ 8.29 (d, *J* = 8.4 Hz, 1H), 8.23 (d, *J* = 8.6 Hz, 1H), 7.96 (t, *J* = 7.8 Hz, 1H), 7.84 - 7.73 (m, 2H), 7.72 - 7.61 (m, 2H), 7.54 (dd, *J* = 8.4, 1.8 Hz, 2H), 5.59 (s, 2H).

3.16 4-((4-fluorobenzyl)oxy)quinoline-2-carboxylic acid (5e)

Intermediate **4e** (2.15 g, 6.91 mmol) and lithium hydroxide (0.83 g, 34.53 mmol) were treated according to the preparation procedure of **5a**. **5e** (1.60 g) was obtained as white solid, yield: 78%, melting point: 272 - 274 °C. ^{1}H NMR (400 MHz, DMSO-d_6) δ 8.16 (dd, *J* = 8.4, 1.4 Hz, 1H), 7.87 (d, *J* = 8.4 Hz, 1H), 7.80 - 7.7ff1 (m, 2H), 7.70 - 7.62 (m, 2H), 7.57 (ddd, *J* = 8.2, 6.8, 1.2 Hz, 1H), 7.37 - 7.25 (m, 2H), 5.46 (s, 2H).

3.17 4-((4-methoxybenzyl)oxy)quinoline-2-carboxylic acid (5f)

Intermediate **4f** (2.23 g, 6.91 mmol) and lithium hydroxide (0.83 g, 34.53 mmol) were treated according to the preparation procedure of **5a**. **5f** (1.70 g) was obtained as white solid, yield: 78%, melting point: > 300 °C. ^{1}H NMR (400 MHz, DMSO-d_6) δ 8.06 (d, *J* = 8.6 Hz, 1H), 7.67 (t, *J* = 7.8 Hz, 1H), 7.56 - 7.41 (m, 4H), 6.99 (d, *J* = 8.3 Hz, 2H), 5.28 (s, 2H), 3.78 (s, 3H).

3.18 4-phenethoxyquinoline-2-carboxylic acid (5g)

Intermediate **4g** (2.12 g, 6.91 mmol) and lithium hydroxide (0.83 g, 34.53 mmol) were treated according to the preparation procedure of **5a**. **5g** (1.30 g) was obtained as white solid, yield: 65%, melting point: 236 - 238 °C. ^{1}H NMR (400 MHz, DMSO-d_6) δ 8.08 (d, *J* = 8.3 Hz, 1H), 7.86 (d, *J* = 8.5 Hz, 1H), 7.75 (t, *J* = 7.7 Hz, 1H), 7.62 (s, 1H), 7.56 (t, *J* = 7.7 Hz, 1H), 7.43 (d, *J* = 7.5 Hz, 2H), 7.33 (t, *J* = 7.5 Hz, 2H), 7.23 (t, *J* = 7.5 Hz, 1H), 4.54 (t, *J* = 6.7 Hz, 2H), 3.22 (t, *J* = 6.6 Hz, 2H).

3.19 4-([1,1'-biphenyl]-4-ylmethoxy)quinoline-2-carboxylic acid (5h)

Intermediate **4h** (2.55 g, 6.91 mmol) and lithium hydroxide (0.83 g, 34.53 mmol) were treated according to the preparation procedure of **5a**. **5h** (1.50 g) was obtained as white solid, yield: 60%, melting point: > 300 °C. ^{1}H NMR (400 MHz, DMSO-d_6) δ 8.21 (d, *J* = 8.1 Hz, 1H), 7.91 (d, *J* = 8.3 Hz, 1H), 7.79 - 7.72 (m, 4H), 7.69 (d, *J* = 3.2 Hz, 2H), 7.65 - 7.58 (m, 2H), 7.49 (t, *J* = 7.6 Hz, 2H), 7.43 - 7.33 (m, 2H), 5.53 (s, 2H).

3.20 4-(naphthalen-1-ylmethoxy)quinoline-2-carboxylic acid (5i)

Intermediate **4i** (2.37 g, 6.91 mmol) and lithium hydroxide (0.83 g, 34.53 mmol) were treated according to the preparation procedure of **5a**. **5i** (1.37 g) was obtained as

white solid, yield: 80%, melting point: > 300 °C. ^{1}H NMR (400 MHz, DMSO-d_6) δ 8.20 (dd, J = 8.3, 4.3 Hz, 1H), 8.08 (d, J = 8.4 Hz, 1H), 8.01 (t, J = 7.8 Hz, 3H), 7.94 (s, 1H), 7.85 - 7.73 (m, 2H), 7.58 (t, J = 8.2 Hz, 4H), 5.95 (s, 2H).

3.21 Methyl 6-(4-(benzyloxy)quinoline-2-carboxamido)hexanoate (6a)

Under an ice bath, **5b** (0.28 g, 1.00 mmol), methyl 6-aminocaproate hydrochloride (0.18 g, 1.00 mmol) and HATU (0.46 g, 1.20 mmol) were dissolved in DMF. NMM (0.35 ml, 3.00 mmol) was added, and the mixture was stirred for 8 h under nitrogen protection. After the reaction, the mixture was poured into water, and extracted with ethyl acetate (3×20 mL). The combined organic layer was washed with 5% citric acid, saturated $NaHCO_3$ solution and saturated brine. After drying with anhydrous $MgSO_4$, filtered and evaporated to dryness, a crude product was obtained. Finally, **6a** (0.32 g) was get after purification by recrystallization with ethyl acetate. Yield: 79%, colorless oil. ^{1}H NMR (400 MHz, DMSO-d_6) δ 8.90 (t, J = 6.2 Hz, 1H), 8.24 (dd, J = 8.5, 1.4 Hz, 1H), 8.08 (d, J = 8.4 Hz, 1H), 7.85 (ddd, J = 8.5, 6.8, 1.5 Hz, 1H), 7.72 (s, 1H), 7.67 (ddd, J = 8.2, 6.9, 1.2 Hz, 1H), 7.62 - 7.57 (m, 2H), 7.49 - 7.44 (m, 2H), 7.42 - 7.37 (m, 1H), 5.51 (s, 2H), 3.57 (s, 3H), 2.32 (t, J = 7.4 Hz, 2H), 1.61 - 1.54 (m, 4H), 1.45 - 1.12 (m, 4H).

3.22 Methyl 6-(4-phenethoxyquinoline-2-carboxamido)hexanoate (6b)

5g (0.29 g, 1.00 mmol), methyl 6-aminocaproate hydrochloride (0.18 g, 1.00 mmol), HATU (0.46 g, 1.20 mmol) and NMM (0.35 ml, 3.00 mmol) were treated according to the preparation procedure of **6a**. **6b** (0.27 g) was purified by recrystallization with alcohol and obtained as white solid, yield: 65%, melting point: 156 - 158 °C. ^{1}H NMR (400 MHz, DMSO-d_6) δ 8.86 (t, J = 6.2 Hz, 1H), 8.13 (dd, J = 8.4, 1.4 Hz, 1H), 8.04 (d, J = 8.4 Hz, 1H), 7.82 (ddd, J = 8.4, 6.8, 1.5 Hz, 1H), 7.68 - 7.59 (m, 2H), 7.47 - 7.40 (m, 2H), 7.34 (t, J = 7.5 Hz, 2H), 7.29 - 7.20 (m, 1H), 4.57 (t, J = 6.4 Hz, 2H), 3.57 (s, 3H), 3.22 (t, J = 6.3 Hz, 2H), 2.31 (t, J = 7.4 Hz, 2H), 1.57 (pd, J = 7.5, 2.3 Hz, 4H), 1.52 - 1.11 (m, 4H).

3.23 Methyl 6-(4-([1,1'-biphenyl]-4-ylmethoxy)quinoline-2-carboxamido) hexanoate (6c)

5h (0.34 g, 1.00 mmol), methyl 6-aminocaproate hydrochloride (0.18 g, 1.00 mmol), HATU (0.46 g, 1.20 mmol) and NMM (0.35 ml, 3.00 mmol) were treated according to the preparation procedure of **6a**. **6c** (0.30 g) was purified by column chromatography with ethyl acetate and petroleum ether (ethyl acetate: petroleum ether = 10:1) and obtained as yellowish oil, yield: 62%. ^{1}H NMR (400 MHz, DMSO-d_6) δ

8.91 (t, *J* = 6.2 Hz, 1H), 8.27 (d, *J* = 8.3 Hz, 1H), 8.09 (d, *J* = 8.4 Hz, 1H), 7.86 (ddd, *J* = 8.8, 7.0, 1.6 Hz, 2H), 7.77 - 7.74 (m, 3H), 7.69 (dd, *J* = 10.9, 7.9 Hz, 6H), 7.43 - 7.34 (m, 2H), 5.56 (s, 2H), 3.57 (s, 3H), 2.32 (t, *J* = 7.4 Hz, 2H), 1.58 (td, *J* = 7.5, 4.1 Hz, 4H), 1.52 - 1.08 (m, 4H).

3.24 Methyl 7-(4-((4-(tert-butyl)benzyl)oxy)quinoline-2-carboxamido)heptanoate (6d)

5c (0.34 g, 1.00 mmol), methyl 7-aminoheptanoate hydrochloride (0.19 g, 1.00 mmol), HATU (0.46 g, 1.20 mmol) and NMM (0.35 ml, 3.00 mmol) were treated according to the preparation procedure of **6a**. **6d** (0.31 g) was purified by column chromatography with ethyl acetate and petroleum ether (ethyl acetate : petroleum ether = 5:1) and obtained as brown oil, yield: 65%. ^{1}H NMR (400 MHz, DMSO-d_6) δ 8.88 (t, *J* = 6.2 Hz, 1H), 8.25 - 8.21 (m, 1H), 8.07 (d, *J* = 8.4 Hz, 1H), 7.85 (ddd, *J* = 8.5, 6.9, 1.5 Hz, 1H), 7.72 (s, 1H), 7.68 - 7.63 (m, 1H), 7.53 - 7.46 (m, 4H), 5.46 (s, 2H), 3.57 (s, 3H), 2.32 (t, *J* = 7.4 Hz, 2H), 1.59 (dp, *J* = 11.1, 3.0 Hz, 4H), 1.30 (s, 13H).

3.25 Methyl 7-(4-((4-chlorobenzyl)oxy)quinoline-2-carboxamido)heptanoate (6e)

5b (0.31 g, 1.00 mmol), methyl 7-aminoheptanoate hydrochloride (0.19 g, 1.00 mmol), HATU (0.46 g, 1.20 mmol) and NMM (0.35 ml, 3.00 mmol) were treated according to the preparation procedure of **6a**. **6e** (0.32 g) was purified by recrystallization with alcohol and obtained as yellowish solid, yield: 70%, melting point: 80 - 82 °C. ^{1}H NMR (400 MHz, DMSO-d_6) δ 8.88 (t, *J* = 5.9 Hz, 1H), 8.24 (d, *J* = 8.3 Hz, 1H), 8.08 (d, *J* = 8.4 Hz, 1H), 7.89 - 7.83 (m, 1H), 7.70 (s, 1H), 7.70 - 7.66 (m, 1H), 7.62 (d, *J* = 8.3 Hz, 2H), 7.55 - 7.49 (m, 2H), 5.51 (s, 2H), 3.57 (s, 3H), 2.28 (dt, *J* = 11.9, 7.3 Hz, 2H), 1.61 - 1.48 (m, 4H), 1.39 - 1.18 (m, 6H).

3.26 Methyl 7-(4-phenethoxyquinoline-2-carboxamido)heptanoate (6f)

5g (0.29 g, 1.00 mmol), methyl 7-aminoheptanoate hydrochloride (0.19 g, 1.00 mmol), HATU (0.46 g, 1.20 mmol) and NMM (0.35 ml, 3.00 mmol) were treated according to the preparation procedure of **6a**. **6f** (0.25 g) was purified by column chromatography with ethyl acetate and petroleum ether (ethyl acetate: petroleum ether = 4:1) and obtained as yellow oil, yield: 58%. ^{1}H NMR (400 MHz, DMSO-d_6) δ 8.85 (t, *J* = 6.2 Hz, 1H), 8.13 (dd, *J* = 8.7, 1.3 Hz, 1H), 8.04 (d, *J* = 8.4 Hz, 1H), 7.82 (ddd, *J* = 8.4, 6.9, 1.5 Hz, 1H), 7.64 (ddd, *J* = 8.2, 6.9, 1.2 Hz, 1H), 7.60 (s, 1H), 7.47 - 7.40 (m, 2H), 7.34 (t, *J* = 7.6 Hz, 2H), 7.27 - 7.20 (m, 1H), 4.57 (t, *J* = 6.4 Hz, 2H), 3.22 (t, *J* = 6.4 Hz, 2H), 2.29 (t, *J* = 7.4 Hz, 2H), 1.72 - 1.53 (m, 4H), 1.52 - 1.14 (m, 6H).

3.27 Methyl 7-(4-([1,1'-biphenyl]-4-ylmethoxy)quinoline-2-carboxamido)

heptanoate (6h)

5h (0.36 g, 1.00 mmol), methyl 7-aminoheptanoate hydrochloride (0.19 g, 1.00 mmol), HATU (0.46 g, 1.20 mmol) and NMM (0.35 ml, 3.00 mmol) were treated according to the preparation procedure of **6a**. **6h** (0.28 g) was purified by column chromatography with ethyl acetate and petroleum ether (ethyl acetate: petroleum ether = 5:1) and obtained as white solid, yield: 55%, melting point: 74 - 76 °C. ^{1}H NMR (400 MHz, DMSO-d_6) δ 8.90 (d, J = 5.9 Hz, 1H), 8.25 (d, J = 8.4 Hz, 1H), 8.09 (d, J = 8.4 Hz, 1H), 7.95 (s, 1H), 7.88 - 7.84 (m, 1H), 7.75 (dd, J = 6.3, 1.9 Hz, 2H), 7.72 - 7.68 (m, 4H), 7.67 (s, 1H), 7.49 (t, J = 7.6 Hz, 2H), 7.41 - 7.38 (m, 1H), 5.56 (s, 2H), 3.56 (s, 3H), 2.33 - 2.26 (m, 2H), 1.54 (dd, J = 15.2, 7.6 Hz, 4H), 1.32 (d, J = 4.0 Hz, 6H).

3.28 Methyl 7-(4-(naphthalen-1-ylmethoxy)quinoline-2-carboxamido)heptanoate (6i)

5i (0.33 g, 1.00 mmol), methyl 6-aminocaproate hydrochloride (0.18 g, 1.00 mmol), HATU (0.46 g, 1.20 mmol) and NMM (0.35 ml, 3.00 mmol) were treated according to the preparation procedure of **6a**. **6i** (0.33 g) was purified by recrystallization with alcohol and obtained as white solid, yield: 70%, melting point: 94 - 96 °C. ^{1}H NMR (400 MHz, DMSO-d_6) δ 8.92 (d, J = 6.4 Hz, 1H), 8.25 - 8.18 (m, 2H), 8.14 - 8.09 (m, 2H), 8.01 (s, 1H), 7.92 (s, 1H), 7.86 - 7.81 (m, 2H), 7.59 (d, J = 8.8 Hz, 4H), 5.97 (s, 2H), 3.57 (s, 3H), 2.33 (d, J = 7.3 Hz, 2H), 1.71 - 1.53 (m, 6H), 1.34 (d, J = 7.1 Hz, 4H).

3.29 4-(benzyloxy)-*N*-(6-(hydroxyamino)-6-oxohexyl)quinoline-2-carboxamide (SDFZ-1)

Hydroxylamine hydrochloride (4.67 g, 67.00 mmol) was dissolved in anhydrous methanol (24 mL) to obtain solution A. Potassium hydroxide (6.60 g, 100.00 mmol) was dissolved in anhydrous methanol (14 mL) to obtain solution B. Under the ice bath, the solution B was dropped into solution A, and the mixture was stirred for 30 min after all the drops finished. The solution was filtered, and the liquid was the final solution of potassium hydroxide and hydroxylamine. **6a** (0.20 g, 0.50 mmol) was dissolved in 5 mL potassium hydroxide, hydroxylamine methanol solution and 5 mL DMF. The mixture was stirred overnight. After that, the methanol was vapored and, the residue was adjusted to neutrality with 1 M hydrochloric acid. The deposit was filtered and recrystallized with ethanol to obtain the product **SDFZ-1** (0.13 g). White solid, yield: 25%, melting point: 164 - 166 °C. ^{1}H NMR (400 MHz, DMSO-d_6) δ 10.33 (s, 1H), 8.87 (t, J = 6.0 Hz, 1H), 8.68 - 8.62 (m, 1H), 8.24 (d, J = 7.7 Hz, 1H), 8.08 (d, J = 8.5 Hz,

1H), 7.88 - 7.82 (m, 1H), 7.72 (s, 1H), 7.67 (t, J = 7.6 Hz, 1H), 7.59 (d, J = 7.4 Hz, 2H), 7.42 (dt, J = 27.3, 7.3 Hz, 3H), 5.51 (s, 2H), 1.96 (t, J = 7.3 Hz, 2H), 1.54 (dq, J = 15.2, 7.7 Hz, 4H), 1.30 (d, J = 7.6 Hz, 2H). ^{13}C NMR (101 MHz, DMSO-d_6) δ 169.53, 164.32, 162.38, 152.32, 147.64, 136.52, 131.17, 129.46, 129.10, 128.66, 128.13, 127.71, 122.21, 121.84, 99.60, 70.57, 33.34, 32.68, 29.48, 26.55, 25.39. HRMS (ESI$^+$): *m/z* calculated for $C_{23}H_{25}N_3O_4$ 408.1918, found [M + H]$^+$ 408.1884.

3.30 *N*-(6-(hydroxyamino)-6-oxohexyl)-4-phenethoxyquinoline-2-carboxamide (SDFZ-2)

6b (0.21 g, 0.50 mmol) was dissolved in 5 mL potassium hydroxide, hydroxylamine methanol solution and 5 mL DMF, then were treated according to **SDFZ-1** to give **SDFZ-2** (0.06 g) as white solid, yield: 30%, melting point: 156 - 157 °C. ^{1}H NMR (400 MHz, DMSO-d_6) δ 10.35 (s, 1H), 8.87 (d, J = 8.0 Hz, 1H), 8.68 (s, 1H), 8.28 - 8.02 (m, 3H), 7.86 - 7.81 (m, 1H), 7.71 - 7.59 (m, 4H), 7.52 (d, J = 8.2 Hz, 1H), 7.43 (d, J = 7.6 Hz, 2H), 7.35 (d, J = 7.4 Hz, 1H), 7.23 (t, J = 7.3 Hz, 1H), 5.51 (s, 2H), 4.56 (d, J = 7.9 Hz, 2H), 3.22 (t, J = 6.5 Hz, 2H), 1.98 - 1.91 (m, 2H), 1.53 (dd, J = 15.2, 7.5 Hz, 4H), 1.30 (s, 4H). ^{13}C NMR (201 MHz, DMSO-d_6) δ 169.51, 164.35, 162.46, 152.31, 151.17, 147.64, 133.48, 131.14, 129.45, 128.11, 127.66, 125.84, 122.21, 121.85, 99.50, 70.44, 34.81, 32.68, 31.58, 29.49, 26.56, 25.40. HRMS (ESI$^+$): *m/z* calculated for $C_{24}H_{27}N_3O_4$ 422.2074, found [M + H]$^+$ 422.2035.

3.31 4-([1,1'-biphenyl]-4-ylmethoxy)-*N*-(6-(hydroxyamino)-6-oxohexyl)quinoline-2-carboxamide (SDFZ-3)

6c (0.24 g, 0.50 mmol) was dissolved in 5 mL potassium hydroxide, hydroxylamine methanol solution and 5 mL DMF, then were treated according to **SDFZ-1** to give **SDFZ-3** (0.04 g) as white solid, yield: 18%, melting point: 154 - 156 °C. ^{1}H NMR (400 MHz, DMSO-d_6) δ 10.37 (s, 1H), 8.90 (t, J = 6.2 Hz, 1H), 8.27 (dd, J = 8.4, 1.4 Hz, 1H), 8.09 (d, J = 8.4 Hz, 1H), 7.88 - 7.84 (m, 1H), 7.77 - 7.74 (m, 3H), 7.72 - 7.66 (m, 5H), 7.49 (t, J = 7.6 Hz, 2H), 7.39 (t, J = 7.3 Hz, 1H), 5.56 (s, 3H), 1.96 (t, J = 7.3 Hz, 2H), 1.56 (dp, J = 23.5, 7.5 Hz, 5H), 1.39 - 1.22 (m, 3H). ^{13}C NMR (101 MHz, DMSO-d_6) δ 173.76, 169.49, 164.34, 162.40, 152.33, 147.67, 140.53, 140.22, 135.69, 131.19, 129.46, 128.78, 128.06, 127.73, 127.41, 127.20, 122.22, 121.86, 107.71, 99.63, 70.31, 32.70, 29.49, 26.56, 25.40. HRMS (ESI$^+$): *m/z* calculated for $C_{29}H_{29}N_3O_4$ 484.2231, found [M + H]$^+$ 484.2202.

3.32 4-((4-(*tert*-butyl)benzyl)oxy)-*N*-(7-(hydroxyamino)-7-oxoheptyl)quinoline-2-carboxamide (SDFZ-4)

6d (0.24 g, 0.50 mmol) was dissolved in 5 mL potassium hydroxide, hydroxylamine methanol solution and 5 mL DMF, then were treated according to **SDFZ-1** to give **SDFZ-4** (0.08 g) as white solid, yield: 35%, melting point: 234 - 236 °C. ^{1}H NMR (400 MHz, DMSO-d_6) δ 10.34 (s, 1H), 8.89 (t, J = 6.1 Hz, 1H), 8.67 (s, 1H), 8.22 (d, J = 8.2 Hz, 1H), 8.08 (d, J = 8.4 Hz, 1H), 7.85 (t, J = 7.5 Hz, 1H), 7.72 (s, 1H), 7.66 (t, J = 7.6 Hz, 1H), 7.49 (q, J = 8.3 Hz, 4H), 5.46 (s, 2H), 1.96 (t, J = 7.3 Hz, 2H), 1.54 (dq, J = 15.4, 7.7 Hz, 4H), 1.30 (s, 9H), 1.28 - 1.02 (m, 4H). ^{13}C NMR (201 MHz, DMSO-d_6) δ 175.99, 169.48, 164.34, 162.47, 162.08, 152.33, 147.57, 138.71, 131.10, 129.93, 129.55, 129.43, 128.84, 127.56, 126.88, 122.04, 121.70, 99.01, 69.69, 35.07, 32.67, 29.49, 26.55, 25.40, 23.99. HRMS (ESI^{+}): *m/z* calculated for $C_{29}H_{29}N_3O_4$ 464.2544, found [M + H]$^{+}$ 464.2514.

3.33 4-((4-chlorobenzyl)oxy)-*N*-(7-(hydroxyamino)-7-oxoheptyl)quinoline-2-carboxamide (SDFZ-5)

6e (0.23 g, 0.50 mmol) was dissolved in 5 mL potassium hydroxide, hydroxylamine methanol solution and 5 mL DMF, then were treated according to **SDFZ-1** to give **SDFZ-5** (0.06 g) as white solid, yield: 27%, melting point: 138 - 140 °C. ^{1}H NMR (400 MHz, DMSO-d_6) δ 10.33 (d, J = 3.1 Hz, 1H), 8.89 (t, J = 6.1 Hz, 1H), 8.24 (dd, J = 8.4, 1.4 Hz, 1H), 8.08 (d, J = 8.4 Hz, 1H), 7.86 (ddd, J = 8.4, 6.8, 1.5 Hz, 1H), 7.73 - 7.61 (m, 4H), 7.59 - 7.49 (m, 2H), 5.51 (s, 2H), 1.59 - 1.46 (m, 4H), 1.35 - 1.21 (m, 6H). ^{13}C NMR (101 MHz, DMSO-d_6) δ 164.30, 162.23, 153.12, 152.31, 147.63, 135.57, 133.25, 131.19, 129.97, 129.46, 129.10, 127.72, 122.18, 121.76, 99.61, 69.74, 35.87, 32.70, 29.60, 28.81, 26.71, 25.55. HRMS (ESI^{+}): *m/z* calculated for $C_{24}H_{26}ClN_3O_4$ 456.1685, found [M + H]$^{+}$ 456.1686.

3.34 4-([1,1'-biphenyl]-4-ylmethoxy)-*N*-(7-(hydroxyamino)-7-oxoheptyl) quinoline-2-carboxamide (SDFZ-6)

6f (0.25 g, 0.50 mmol) was dissolved in 5 mL potassium hydroxide, hydroxylamine methanol solution and 5 mL DMF, then were treated according to **SDFZ-1** to give **SDFZ-6** (0.06 g) as white solid, yield: 25%, melting point: 200 - 202 °C. ^{1}H NMR (400 MHz, DMSO-d_6) δ 8.89 (s, 1H), 8.27 (d, J = 8.3 Hz, 1H), 8.09 (d, J = 8.4 Hz, 1H), 7.88 - 7.83 (m, 1H), 7.75 (t, J = 4.0 Hz, 2H), 7.69 (dd, J = 11.5, 7.8 Hz, 4H), 7.49 (t, J = 7.4 Hz, 3H), 7.39 (d, J = 7.4 Hz, 2H), 5.56 (s, 2H), 2.98 (s, 2H), 2.03 (d, J = 8.0 Hz, 1H), 1.93 (d, J = 8.0 Hz, 1H), 1.56 - 1.45 (m, 4H), 1.30 (s, 4H). ^{13}C NMR (101 MHz, DMSO-d_6) δ 162.38, 159.45, 152.32, 147.77, 140.19, 136.26, 135.79, 135.66, 131.17, 129.56, 129.48, 129.44, 128.84, 128.76, 128.07, 128.04, 127.69,

127.39, 127.17, 121.85, 99.61, 70.31, 68.50, 29.61, 28.90, 26.73. HRMS (ESI^+): *m/z* calculated for $C_{30}H_{31}N_3O_4$ 498.2387, found $[M + H]^+$ 498.2386.

3.35 *N*-(7-(hydroxyamino)-7-oxoheptyl)-4-(naphthalen-1-ylmethoxy)quinoline-2-carboxamide (SDFZ-7)

6h (0.23 g, 0.50 mmol) was dissolved in 5 mL potassium hydroxide, hydroxylamine methanol solution and 5 mL DMF, then were treated according to **SDFZ-1** to give **SDFZ-7** (0.03 g) as white solid, yield: 15%, melting point: 224 - 226 °C. ^1H NMR (400 MHz, DMSO-d_6) δ 10.35 (s, 1H), 8.91 (s, 1H), 8.20 (s, 1H), 8.10 (dd, *J* = 12.8, 8.3 Hz, 2H), 8.01 (t, *J* = 7.8 Hz, 2H), 7.92 (s, 1H), 7.85 - 7.79 (m, 2H), 7.73 (s, 1H), 7.59 (q, *J* = 8.7 Hz, 3H), 5.97 (s, 2H), 2.03 - 1.93 (m, 2H), 1.54 (d, *J* = 35.5 Hz, 4H), 1.31 (s, 4H). ^{13}C NMR (101 MHz, DMSO-d_6) δ 166.59, 164.78, 162.31, 151.99, 147.71, 145.63, 135.56, 133.27, 131.29, 129.99, 129.74, 129.11, 128.02, 122.24, 121.88, 99.76, 69.79, 52.53. HRMS (ESI^+): *m/z* calculated for $C_{28}H_{29}N_3O_4$ 472.2231, found $[M + H]^+$ 472.2227.

3.36 *N*-(7-(hydroxyamino)-7-oxoheptyl)-4-phenethoxyquinoline-2-carboxamide (SDFZ-8)

6i (0.22 g, 0.50 mmol) was dissolved in 5 mL potassium hydroxide, hydroxylamine methanol solution and 5 mL DMF, then were treated according to **SDFZ-1** to give **SDFZ-8** (0.11 g) as white solid, yield: 48%, melting point: 118 - 120 °C. ^1H NMR (400 MHz, DMSO-d_6) δ 10.35 (s, 1H), 8.87 (t, *J* = 6.1 Hz, 1H), 8.71 (s, 1H), 8.19 - 8.10 (m, 1H), 8.05 (d, *J* = 8.4 Hz, 1H), 7.82 (ddd, *J* = 8.5, 6.9, 1.5 Hz, 1H), 7.69 - 7.58 (m, 2H), 7.43 (d, *J* = 7.1 Hz, 2H), 7.34 (t, *J* = 7.5 Hz, 2H), 7.24 (t, *J* = 7.3 Hz, 1H), 4.57 (t, *J* = 6.4 Hz, 2H), 3.22 (t, *J* = 6.4 Hz, 2H), 1.94 (t, *J* = 7.4 Hz, 2H), 1.54 (dq, *J* = 26.8, 7.1, 6.7 Hz, 4H), 1.30 (qd, *J* = 7.8, 5.1, 4.4 Hz, 4H). ^{13}C NMR (101 MHz, DMSO-d_6) δ 164.34, 162.47, 152.36, 147.58, 138.70, 131.08, 129.54, 129.43, 128.84, 127.55, 126.88, 122.04, 121.71, 99.01, 69.69, 35.07, 32.68, 29.62, 28.82, 26.68, 25.56. HRMS (ESI^+): *m/z* calculated for $C_{25}H_{29}N_3O_4$ 436.2231, found $[M + H]^+$ 436.2219. (**Figure S9-S11**)

3.37 *tert*-Butyl (4-bromobenzyl)carbamate (8a)

4-bromobenzylamine (10.00 g, 53.75 mmol) and triethylamine (10.88 g, 107.5 mmol) were dissolved in 100 mL of dichloromethane, and di-*tert*-butyl dicarbonate (11.73 g, 53.75 mmol) was added dropwise. After stirring for 2 h, dichloromethane was vapored, and ethyl acetate was added and washed with saturated $NaHCO_3$ solution for three times. After dried with anhydrous $MgSO_4$, ethyl acetate was vapored to give the

product **8a** (14.77 g). White solid, yield: 96%, melting point: 85 - 86 ℃. ^{1}H NMR (400 MHz, DMSO-d_6) δ 7.51 (d, *J* = 7.9 Hz, 2H), 7.21 - 7.17 (m, 2H), 4.09 (d, *J* = 6.2 Hz, 2H), 1.39 (s, 9H).

3.38 *tert*-Butyl (3-bromobenzyl)carbamate (8b)

3-bromobenzylamine (10.00 g, 53.75 mmol) and triethylamine (10.88 g, 107.5 mmol) were treated with di-*tert*-butyl dicarbonate (11.73 g, 53.75 mmol) according to the preparation procedure of **8a** to give **8b** (15.22 g) as white solid, yield: 99%, melting point: 85 - 86 ℃. ^{1}H NMR (400 MHz, DMSO-d_6) δ 7.50 - 7.42 (m, 2H), 7.28 (dd, *J* = 16.1, 8.5 Hz, 2H), 4.12 (d, *J* = 6.3 Hz, 2H), 1.39 (s, 9H).

3.39 Methyl (*E*)-3-(4-(((tert-butoxycarbonyl)amino)methyl)phenyl)acrylate (9a)

8a (10 g, 34.94 mmol), methyl acrylate (9.03 g, 104.80 mmol), triethylamine (10.61 g, 104.83 mmol), and tetrakistriphenylphosphine palladium (0.10 g) were dissolved in 50 mL DMF and heated to 130°C for 12 h. After cooled to room temperature naturally, the mixture was quenched by 300 mL ice water and extracted 3 times with ethyl acetate. The combined organic layer was washed with 5% citric acid, saturated $NaHCO_3$ solution and saturated brine. After drying with anhydrous $MgSO_4$, filtered and evaporated to dryness, a crude product was obtained. Then the crude product was purified by column chromatography with ethyl acetate and petroleum ether (ethyl acetate: petroleum ether = 5:1) to give **9a** (7.63 g). White solid, yield: 75%, melting point: 66 - 68 ℃. ^{1}H NMR (400 MHz, DMSO-d_6) δ 7.69 - 7.61 (m, 3H), 7.44 (t, *J* = 6.1 Hz, 1H), 7.27 (d, *J* = 7.9 Hz, 2H), 6.61 (d, *J* = 16.0 Hz, 1H), 4.14 (d, *J* = 6.2 Hz, 2H), 3.72 (s, 3H), 1.39 (s, 9H).

3.40 Methyl (*E*)-3-(3-(((tert-butoxycarbonyl)amino)methyl)phenyl)acrylate (9b)

8b (10 g, 34.94 mmol), methyl acrylate (9.03 g, 104.80 mmol), triethylamine (10.61 g, 104.83 mmol), and tetrakistriphenylphosphine palladium (0.10 g) were reacted according to the preparation procedure of **8a** to give **8b** (6.61 g) as white solid, yield: 65%, melting point: 90 - 92 ℃. ^{1}H NMR (400 MHz, DMSO-d_6) δ 7.70 - 7.54 (m, 3H), 7.47 - 7.27 (m, 3H), 6.60 (d, *J* = 16.0 Hz, 1H), 4.15 (d, *J* = 6.2 Hz, 2H), 3.73 (s, 3H), 1.40 (s, 9H).

3.41 (*E*)-(4-(3-methoxy-3-oxoprop-1-en-1-yl)phenyl)methanamine hydrochloride (10a)

9a (8.7 g, 30 mmol) was dissolved in a saturated ethyl acetate solution of hydrogen chloride, and the mixture was sealed and stirred for 2 h. The precipitate was filtered, washed with ethyl acetate and dried to give **10a** (6.47 g). White solid, yield: 95%,

melting point: 240 - 242 °C. ^{1}H NMR (400 MHz, DMSO-d_6) δ 8.47 (s, 3H), 7.81 - 7.75 (m, 2H), 7.67 (d, *J* = 16.1 Hz, 1H), 7.60 - 7.49 (m, 2H), 6.70 (d, *J* = 16.1 Hz, 1H), 4.05 (s, 2H), 3.73 (s, 3H).

3.42 (*E*)-(3-(3-methoxy-3-oxoprop-1-en-1-yl)phenyl)methanaminium chloride (10b)

9b (8.7 g, 30 mmol) was dissolved in a saturated ethyl acetate solution of hydrogen chloride, and the mixture was sealed and stirred for 2 h. The precipitate was filtered, washed with ethyl acetate and dried to give **10b** (6.13 g). White solid, yield: 90%, melting point: 216 - 218 °C. ^{1}H NMR (400 MHz, DMSO-d_6) δ 8.52 (s, 3H), 7.97 - 7.92 (m, 1H), 7.71 (d, *J* = 7.9 Hz, 1H), 7.66 (d, *J* = 16.0 Hz, 1H), 7.56 (d, *J* = 7.6 Hz, 1H), 7.47 (t, *J* = 7.7 Hz, 1H), 6.68 (d, *J* = 16.0 Hz, 1H), 4.05 (s, 2H), 3.74 (s, 3H).

3.43 Methyl (*E*)-3-(4-((4-methoxyquinoline-2-carboxamido)methyl)phenyl) acrylate (11a)

Under an ice bath, **5a** (0.20 g, 1.00 mmol), **10a** (0.23 g, 1.00 mmol) and HATU (0.46 g, 1.20 mmol) were dissolved in DMF. NMM (0.35 ml, 3.00 mmol) was added, and the mixture was stirred for 8 h under nitrogen protection. After the reaction, the mixture was poured into water, and extracted with ethyl acetate (3×20 mL). The combined organic layer was washed with 5% citric acid, saturated $NaHCO_3$ solution and saturated brine. After drying with anhydrous $MgSO_4$, filtered and evaporated to dryness, a crude product was obtained. Finally, **11a** (0.26 g) was get after purification by recrystallized with ethanol. White solid, yield: 70%, melting point: >300 °C. ^{1}H NMR (400 MHz, DMSO-d_6) δ 9.50 (t, *J* = 6.4 Hz, 1H), 8.20 (dd, *J* = 8.3, 1.4 Hz, 1H), 8.07 (d, *J* = 8.4 Hz, 1H), 7.87 - 7.83 (m, 1H), 7.75 - 7.66 (m, 4H), 7.63 (d, *J* = 3.1 Hz, 1H), 7.41 (d, *J* = 8.0 Hz, 2H), 6.62 (dd, *J* = 16.1, 4.8 Hz, 1H), 4.58 (d, *J* = 6.4 Hz, 2H), 4.14 (s, 3H), 3.72 (s, 3H).

3.44 Methyl (*E*)-3-(4-((4-(benzyloxy)quinoline-2-carboxamido)methyl)phenyl) acrylate (11b)

5c (0.28 g, 1.00 mmol), **10a** (0.23 g, 1.00 mmol) and HATU (0.46 g, 1.20 mmol) and NMM (0.35 ml, 3 mmol) were reacted according to the preparation procedure of **11a** to give **11b** (0.30 g) as white solid, yield: 66%, melting point: 128 - 130 °C. ^{1}H NMR (400 MHz, DMSO-d_6) δ 9.51 (t, *J* = 6.4 Hz, 1H), 8.25 (dd, *J* = 8.5, 1.4 Hz, 1H), 8.09 (d, *J* = 8.5 Hz, 1H), 7.89 - 7.84 (m, 1H), 7.75 (d, *J* = 4.0 Hz, 1H), 7.68 (s, 3H), 7.63 (d, *J* = 2.8 Hz, 1H), 7.61 - 7.57 (m, 2H), 7.48 - 7.44 (m, 2H), 7.43 - 7.38 (m, 3H),

7.31 (d, J = 8.2 Hz, 1H), 6.63 - 6.59 (m, 1H), 5.52 (s, 2H), 4.58 (d, J = 6.4 Hz, 2H), 3.72 (s, 3H).

3.45 Methyl (*E*)-3-(4-((4-((4-(*tert*-butyl)benzyl)oxy)quinoline-2-carboxamido) methyl)phenyl)acrylate (11c)

5f (0.34 g, 1.00 mmol), **10a** (0.23 g, 1.00 mmol) and HATU (0.46 g, 1.20 mmol) and NMM (0.35 ml, 3 mmol) were reacted according to the preparation procedure of **11a** to give **11c** (0.31 g) as white solid, yield: 60%, melting point: 162 - 164 °C. ^{1}H NMR (400 MHz, DMSO-d_6) δ 9.50 (t, J = 6.4 Hz, 1H), 8.23 (dd, J = 8.6, 1.5 Hz, 1H), 8.08 (d, J = 8.4 Hz, 1H), 7.86 (ddd, J = 8.4, 6.9, 1.5 Hz, 1H), 7.75 (s, 1H), 7.72 - 7.61 (m, 4H), 7.49 (q, J = 8.5 Hz, 4H), 7.41 (d, J = 8.0 Hz, 2H), 6.61 (d, J = 16.0 Hz, 1H), 5.46 (s, 2H), 4.58 (d, J = 6.4 Hz, 2H), 3.72 (s, 3H), 1.30 (s, 9H).

3.46 Methyl (*E*)-3-(4-((4-((4-chlorobenzyl)oxy)quinoline-2-carboxamido)methyl) phenyl)acrylate (11d)

5d (0.31g, 1.00 mmol), **10a** (0.23 g, 1.00 mmol) and HATU (0.46 g, 1.20 mmol) and NMM (0.35 ml, 3 mmol) were reacted according to the preparation procedure of **11a** to give **11d** (0.34 g) as white solid, yield: 70%, melting point: 158 - 160 °C. ^{1}H NMR (400 MHz, DMSO-d_6) δ 9.50 (t, J = 6.4 Hz, 1H), 8.29 - 8.22 (m, 1H), 8.09 (d, J = 8.4 Hz, 1H), 7.86 (ddd, J = 8.5, 6.9, 1.5 Hz, 1H), 7.73 (s, 1H), 7.72 - 7.66 (m, 3H), 7.66 - 7.57 (m, 3H), 7.57 - 7.49 (m, 2H), 7.41 (d, J = 8.0 Hz, 2H), 6.61 (d, J = 16.0 Hz, 1H), 5.52 (s, 2H), 4.58 (d, J = 6.4 Hz, 2H), 3.72 (s, 3H).

3.47 Methyl (*E*)-3-(4-((4-((4-fluorobenzyl)oxy)quinoline-2-carboxamido)methyl) phenyl)acrylate (11e)

5e (0.30 g, 1.00 mmol), **10a** (0.23 g, 1.00 mmol) and HATU (0.46 g, 1.20 mmol) and NMM (0.35 ml, 3 mmol) were reacted according to the preparation procedure of **11a** to give **11e** (0.30 g) as white solid, yield: 64%, melting point: 147 - 149 °C. ^{1}H NMR (400 MHz, DMSO-d_6) δ 9.51 (t, J = 6.4 Hz, 1H), 8.25 - 8.21 (m, 1H), 8.08 (d, J = 8.4 Hz, 1H), 7.95 (s, 1H), 7.86 (ddd, J = 8.4, 6.8, 1.5 Hz, 1H), 7.72 - 7.62 (m, 6H), 7.44 - 7.39 (m, 2H), 7.32 - 7.26 (m, 2H), 6.61 (d, J = 16.1 Hz, 1H), 5.49 (s, 2H), 4.59 (d, J = 6.4 Hz, 2H), 3.72 (s, 3H).

3.48 Methyl (*E*)-3-(4-((4-((4-methoxybenzyl)oxy)quinoline-2-carboxamido)methyl) phenyl)acrylate (11f)

5i (0.31 g, 1.00 mmol), **10a** (0.23 g, 1.00 mmol) and HATU (0.46 g, 1.20 mmol) and NMM (0.35 ml, 3 mmol) were reacted according to the preparation procedure of **11a** and purified by column chromatography with ethyl acetate and petroleum ether

(ethyl acetate: petroleum ether = 5:1) to give **11f** (0.33 g) as brown oil, yield: 68%. ^{1}H NMR (400 MHz, DMSO-d_6) δ 9.48 (t, *J* = 6.4 Hz, 1H), 8.25 (dd, *J* = 8.4, 1.4 Hz, 1H), 8.09 (d, *J* = 8.4 Hz, 1H), 7.86 (ddd, *J* = 8.4, 6.8, 1.5 Hz, 1H), 7.74 (s, 2H), 7.72 - 7.65 (m, 3H), 7.62 (d, *J* = 8.5 Hz, 3H), 7.56 - 7.49 (m, 2H), 7.47 - 7.37 (m, 2H), 6.61 (d, *J* = 16.0 Hz, 1H), 5.51 (s, 2H), 4.59 (d, *J* = 6.4 Hz, 2H), 3.72 (s, 3H).

3.49 Methyl (*E*)-3-(4-((4-phenethoxyquinoline-2-carboxamido)methyl)phenyl) acrylate (11g)

5j (0.29 g, 1.00 mmol), **10a** (0.23 g, 1.00 mmol) and HATU (0.46 g, 1.20 mmol) and NMM (0.35 ml, 3 mmol) were reacted according to the preparation procedure of **11a** to give **11g** (0.26 g) as white solid, yield: 55%, melting point: 118 - 120 °C. ^{1}H NMR (400 MHz, DMSO-d_6) δ 9.47 (t, *J* = 6.4 Hz, 1H), 8.14 (dd, *J* = 8.5, 1.4 Hz, 1H), 8.05 (d, *J* = 8.4 Hz, 1H), 7.83 (ddd, *J* = 8.4, 6.9, 1.5 Hz, 1H), 7.73 - 7.60 (m, 5H), 7.48 - 7.38 (m, 4H), 7.33 (t, *J* = 7.5 Hz, 2H), 7.23 (t, *J* = 7.3 Hz, 1H), 6.61 (d, *J* = 16.0 Hz, 1H), 4.58 (dt, *J* = 6.5, 3.3 Hz, 4H), 3.72 (s, 3H), 3.22 (t, *J* = 6.3 Hz, 2H).

3.50 Methyl (*E*)-3-(3-((4-methoxyquinoline-2-carboxamido)methyl)phenyl) acrylate (11h)

5a (0.20 g, 1.00 mmol), **10b** (0.23 g, 1.00 mmol) and HATU (0.46 g, 1.20 mmol) and NMM (0.35 ml, 3 mmol) were reacted according to the preparation procedure of **11a** and purified by column chromatography with ethyl acetate and petroleum ether (ethyl acetate: petroleum ether = 4:1) to give **11h** (0.17 g) as colorless oil, yield: 45%. ^{1}H NMR (400 MHz, DMSO-d_6) δ 9.49 (t, *J* = 6.4 Hz, 1H), 8.25 (dd, *J* = 8.4, 1.4 Hz, 1H), 8.09 (d, *J* = 8.4 Hz, 1H), 7.86 (ddd, *J* = 8.5, 6.9, 1.5 Hz, 1H), 7.73 (s, 1H), 7.72 - 7.70 (m, 1H), 7.69 - 7.66 (m, 1H), 7.62 (d, *J* = 8.2 Hz, 2H), 7.53 - 7.51 (m, 1H), 7.43 - 7.38 (m, 1H), 6.61 (d, *J* = 16.0 Hz, 1H), 5.52 (s, 3H), 4.59 (d, *J* = 6.4 Hz, 2H), 3.72 (s, 3H).

3.51 Methyl (*E*)-3-(3-((4-((4-(tert-butyl)benzyl)oxy)quinoline-2-carboxamido) methyl)phenyl)acrylate (11i)

5f (0.34 g, 1.00 mmol), **10b** (0.23 g, 1.00 mmol) and HATU (0.46 g, 1.20 mmol) and NMM (0.35 ml, 3 mmol) were reacted according to the preparation procedure of **11a** to give **11i** (0.27 g) as yellow solid, yield: 56%, melting point: 140 - 142 °C. ^{1}H NMR (400 MHz, DMSO-d_6) δ 9.50 (t, *J* = 6.5 Hz, 1H), 8.23 (dd, *J* = 8.4, 1.4 Hz, 1H), 8.11 - 8.06 (m, 1H), 7.85 (ddd, *J* = 8.3, 6.7, 1.5 Hz, 1H), 7.75 (s, 1H), 7.68 (t, *J* = 3.7 Hz, 2H), 7.65 - 7.62 (m, 1H), 7.51 (dd, *J* = 16.5, 7.9 Hz, 4H), 7.41 (d, *J* = 8.0 Hz, 2H),

7.34 - 7.23 (m, 1H), 6.61 (d, *J* = 16.0 Hz, 1H), 5.46 (s, 2H), 4.59 (d, *J* = 6.4 Hz, 2H), 3.72 (s, 3H), 1.30 (s, 9H).

3.52 Methyl (*E*)-3-(3-((4-((4-chlorobenzyl)oxy)quinoline-2-carboxamido)methyl) phenyl)acrylate (11j)

5d (0.31 g, 1.00 mmol), **10b** (0.23 g, 1.00 mmol) and HATU (0.46 g, 1.20 mmol) and NMM (0.35 ml, 3 mmol) were reacted according to the preparation procedure of **11a** to give **11j** (0.29 g) as yellowish solid, yield: 60%, melting point: 150 - 152 °C. ^{1}H NMR (400 MHz, DMSO-d_6) δ 9.47 (t, *J* = 6.4 Hz, 1H), 8.25 (dd, *J* = 8.5, 1.6 Hz, 1H), 8.09 (d, *J* = 8.5 Hz, 1H), 7.86 (ddt, *J* = 8.5, 6.9, 1.6 Hz, 1H), 7.73 (s, 1H), 7.71 - 7.70 (m, 1H), 7.69 - 7.66 (m, 1H), 7.64 - 7.60 (m, 3H), 7.52 (dt, *J* = 8.3, 2.3 Hz, 2H), 7.45 - 7.37 (m, 2H), 6.61 (dd, *J* = 16.0, 1.3 Hz, 1H), 5.51 (d, *J* = 2.0 Hz, 2H), 4.59 (d, *J* = 6.4 Hz, 2H), 3.72 (s, 3H).

3.53 Methyl (*E*)-3-(3-((4-((4-fluorobenzyl)oxy)quinoline-2-carboxamido)methyl) phenyl)acrylate (11k)

5e (0.30 g, 1.00 mmol), **10b** (0.23 g, 1.00 mmol) and HATU (0.46 g, 1.20 mmol) and NMM (0.35 ml, 3 mmol) were reacted according to the preparation procedure of **11a** to give **11k** (0.21 g) as white solid, yield: 45%, melting point: 104 - 106 °C. ^{1}H NMR (400 MHz, DMSO-d_6) δ 9.49 (t, *J* = 6.4 Hz, 1H), 8.23 (dd, *J* = 8.4, 1.4 Hz, 1H), 8.09 (d, *J* = 8.4 Hz, 1H), 7.96 (s, 1H), 7.86 (ddd, *J* = 8.4, 6.8, 1.5 Hz, 1H), 7.75 (s, 1H), 7.72 - 7.60 (m, 6H), 7.47 - 7.37 (m, 2H), 7.33 - 7.25 (m, 2H), 6.61 (d, *J* = 16.1 Hz, 1H), 5.49 (s, 2H), 4.60 (d, *J* = 6.4 Hz, 2H), 3.72 (s, 3H).

3.54 Methyl (*E*)-3-(3-((4-phenethoxyquinoline-2-carboxamido)methyl)phenyl) acrylate (11l)

5g (0.29 g, 1.00 mmol), **10b** (0.23 g, 1.00 mmol) and HATU (0.46 g, 1.20 mmol) and NMM (0.35 ml, 3 mmol) were reacted according to the preparation procedure of **11a** to give **11l** (0.19 g) as white solid, yield: 42%, melting point: 116 - 118 °C. ^{1}H NMR (400 MHz, DMSO-d_6) δ 9.47 (t, *J* = 6.4 Hz, 1H), 8.14 (dd, *J* = 8.3, 1.4 Hz, 1H), 8.05 (d, *J* = 8.4 Hz, 1H), 7.83 (ddd, *J* = 8.4, 6.9, 1.5 Hz, 1H), 7.67 (dd, *J* = 7.7, 5.8 Hz, 3H), 7.62 (d, *J* = 1.9 Hz, 1H), 7.53 - 7.37 (m, 4H), 7.32 (d, *J* = 7.7 Hz, 1H), 7.28 - 7.19 (m, 1H), 6.61 (d, *J* = 16.1 Hz, 1H), 4.59 (d, *J* = 6.2 Hz, 2H), 3.71 (s, 3H), 3.22 (t, *J* = 6.3 Hz, 2H).

3.55 (*E*)-*N*-(4-(3-(hydroxyamino)-3-oxoprop-1-en-1-yl)benzyl)-4-methoxyquinoline-2-carboxamide (SDFZ-9)

11a (0.19 g, 0.50 mmol) was dissolved in 5 mL potassium hydroxide, hydroxylamine methanol solution and 5 mL DMF, then were treated according to **SDFZ-1** to give **SDFZ-9** (0.07 g) as white solid, yield: 35%, melting point: 158 - 160 °C. ^{1}H NMR (400 MHz, DMSO-d_6) δ 9.62 (t, *J* = 6.4 Hz, 1H), 8.22 (dd, *J* = 8.3, 1.4 Hz, 1H), 8.12 (d, *J* = 8.4 Hz, 1H), 7.94 - 7.87 (m, 1H), 7.70 (d, *J* = 2.0 Hz, 1H), 7.57 (d, *J* = 16.0 Hz, 1H), 7.41 (d, *J* = 8.0 Hz, 1H), 6.50 (d, *J* = 16.0 Hz, 1H), 4.59 (d, *J* = 6.3 Hz, 2H), 4.17 (s, 3H). ^{13}C NMR (101 MHz, DMSO-d_6) δ 168.04, 164.75, 158.84, 144.15, 139.48, 131.55, 128.71, 128.40, 128.16, 127.91, 122.19, 120.56, 119.26, 110.32, 98.84, 77.21, 57.08, 50.48, 42.95. HRMS (ESI$^+$): *m/z* calculated for $C_{21}H_{19}N_3O_4$ 378.1448, found $[M + H]^+$ 378.1455.

3.56 (*E*)-4-(benzyloxy)-*N*-(4-(3-(hydroxyamino)-3-oxoprop-1-en-1-yl)benzyl) quinoline-2-carboxamide (SDFZ-10)

11b (0.22 g, 0.50 mmol) was dissolved in 5 mL potassium hydroxide, hydroxylamine methanol solution and 5 mL DMF, then were treated according to **SDFZ-1** to give **SDFZ-10** (0.07 g) as white solid, yield: 30%, melting point: 190 - 192 °C. ^{1}H NMR (400 MHz, DMSO-d_6) δ 9.43 (s, 1H), 8.25 (d, *J* = 8.3 Hz, 1H), 8.08 (d, *J* = 8.5 Hz, 1H), 7.95 (s, 1H), 7.86 (t, *J* = 7.6 Hz, 1H), 7.75 (s, 1H), 7.68 (t, *J* = 7.6 Hz, 1H), 7.59 (d, *J* = 7.4 Hz, 2H), 7.52 - 7.25 (m, 6H), 7.11 (d, *J* = 16.3 Hz, 1H), 6.37 (d, *J* = 15.5 Hz, 1H), 5.52 (s, 2H), 4.55 (d, *J* = 6.2 Hz, 2H). ^{13}C NMR (101 MHz, DMSO-d_6) δ 164.57, 162.41, 152.13, 147.67, 136.49, 131.27, 131.20, 129.71, 129.63, 129.48, 129.28, 129.08, 128.65, 128.37, 128.13, 127.76, 127.13, 122.20, 121.89, 99.72, 71.76, 70.76, 70.58. HRMS (ESI$^+$): *m/z* calculated for $C_{27}H_{23}N_3O_4$ 454.1761, found $[M + H]^+$ 454.1772.

3.57 (*E*)-4-((4-(tert-butyl)benzyl)oxy)-*N*-(4-(3-(hydroxyamino)-3-oxoprop-1-en-1-yl)benzyl)quinoline-2-carboxamide (SDFZ-11)

11c (0.26 g, 0.50 mmol) was dissolved in 5 mL potassium hydroxide, hydroxylamine methanol solution and 5 mL DMF, then were treated according to **SDFZ-1** to give **SDFZ-11** (0.07 g) as white solid, yield: 28%, melting point: 234 - 236 °C。 ^{1}H NMR (400 MHz, DMSO-d_6) δ 9.45 (d, *J* = 10.8 Hz, 1H), 8.22 (d, *J* = 7.6 Hz, 1H), 8.07 (q, *J* = 6.7, 5.6 Hz, 1H), 7.83 (q, *J* = 8.2, 7.2 Hz, 1H), 7.75 (q, *J* = 4.4 Hz, 1H), 7.66 (t, *J* = 7.4 Hz, 1H), 7.56 - 7.28 (m, 9H), 6.43 (dd, *J* = 23.0, 16.0 Hz, 1H), 5.45 (t, *J* = 6.6 Hz, 2H), 4.67 - 4.47 (m, 2H), 1.29 (d, *J* = 4.0 Hz, 9H). ^{13}C NMR (101 MHz, DMSO-d_6) δ 164.55, 162.52, 152.10, 151.20, 147.69, 133.46, 131.21, 129.47, 128.43,

128.37, 128.28, 128.12, 127.91, 127.82, 127.76, 125.96, 125.85, 125.62, 125.53, 121.93, 99.63, 70.48, 42.87, 34.82, 31.58. HRMS (ESI$^+$): *m/z* calculated for $C_{31}H_{31}N_3O_4$ 510.2387, found [M + H]$^+$ 510.2383.

3.58 (*E*)-4-((4-chlorobenzyl)oxy)-*N*-(4-(3-(hydroxyamino)-3-oxoprop-1-en-1-yl)benzyl)quinoline-2-carboxamide (SDFZ-12)

11d (0.24 g, 0.50 mmol) was dissolved in 5 mL potassium hydroxide, hydroxylamine methanol solution and 5 mL DMF, then were treated according to **SDFZ-1** to give **SDFZ-12** (0.08 g) as white solid, yield: 32%, melting point: 244 - 246 °C. ^{1}H NMR (400 MHz, DMSO-d_6) δ 10.21 (s, 1H), 9.45 (d, *J* = 16.2 Hz, 1H), 8.25 (d, *J* = 8.5 Hz, 1H), 8.08 (d, *J* = 8.8 Hz, 1H), 7.86 (t, *J* = 7.6 Hz, 1H), 7.82 - 7.58 (m, 5H), 7.58 - 7.42 (m, 4H), 7.35 (dd, *J* = 15.3, 8.0 Hz, 2H), 6.44 (d, *J* = 16.5 Hz, 1H), 5.52 (s, 2H), 4.56 (d, *J* = 6.4 Hz, 2H). ^{13}C NMR (101 MHz, DMSO-d_6) δ 187.68, 166.86, 165.92, 130.01, 129.12, 128.97, 128.91, 127.34, 99.76, 80.96, 70.05.HRMS (ESI$^+$): *m/z* calculated for $C_{27}H_{22}ClN_3O_4$ 488.1372, found [M + H]$^+$488.1422.

3.59 (*E*)-4-((4-fluorobenzyl)oxy)-*N*-(4-(3-(hydroxyamino)-3-oxoprop-1-en-1-yl)benzyl)quinoline-2-carboxamide (SDFZ-13)

11e (0.24 g, 0.50 mmol) was dissolved in 5 mL potassium hydroxide, hydroxylamine methanol solution and 5 mL DMF, then were treated according to **SDFZ-1** to give **SDFZ-13** (0.09 g) as white solid, yield: 36%, melting point:294 - 296 °C. ^{1}H NMR (400 MHz, DMSO-d_6) δ 9.57 (t, *J* = 6.3 Hz, 1H), 8.24 (dd, *J* = 8.3, 1.5 Hz, 1H), 8.11 (d, *J* = 8.4 Hz, 1H), 7.88 (ddd, *J* = 8.5, 6.9, 1.5 Hz, 1H), 7.79 (s, 1H), 7.74 - 7.61 (m, 5H), 7.57 (d, *J* = 16.0 Hz, 1H), 7.41 (d, *J* = 8.1 Hz, 2H), 7.29 (td, *J* = 8.6, 2.1 Hz, 2H), 6.50 (d, *J* = 16.0 Hz, 1H), 4.59 (d, *J* = 6.4 Hz, 2H). ^{13}C NMR (101 MHz, DMSO-d_6) δ 189.60, 168.04, 163.87, 161.30, 151.30, 144.13, 142.06, 133.40, 132.50, 132.46, 132.06, 131.95, 130.70, 128.79, 128.71, 128.42, 128.31, 128.25, 128.17, 122.46, 122.40, 121.75, 119.28, 116.12, 115.87, 100.21, 70.41, 62.98. HRMS (ESI$^+$): *m/z* calculated for $C_{27}H_{22}FN_3O_4$ 472.1667, found [M + H]$^+$ 472.1692.

3.60 (*E*)-*N*-(4-(3-(hydroxyamino)-3-oxoprop-1-en-1-yl)benzyl)-4-((4-methoxybenzyl)oxy)quinoline-2-carboxamide (SDFZ-14)

11f (0.24 g, 0.50 mmol) was dissolved in 5 mL potassium hydroxide, hydroxylamine methanol solution and 5 mL DMF, then were treated according to **SDFZ-1** to give **SDFZ-14** (0.11 g) as white solid, yield: 45%, melting point:188 - 189 °C. ^{1}H NMR (400 MHz, DMSO-d_6) δ 9.59 (t, *J* = 6.5 Hz, 1H), 8.28 - 8.23 (m, 1H), 8.09 (d, *J* = 8.5 Hz, 1H), 7.99 - 7.91 (m, 2H), 7.87 (ddd, *J* = 8.4, 6.8, 1.5 Hz, 2H), 7.73

(s, 1H), 7.69 (ddt, *J* = 9.9, 7.2, 2.2 Hz, 2H), 7.62 (d, *J* = 8.3 Hz, 2H), 7.53 (d, *J* = 2.0 Hz, 1H), 7.50 (dd, *J* = 8.8, 2.2 Hz, 2H), 7.34 (dd, *J* = 31.3, 7.8 Hz, 1H), 5.52 (s, 2H), 4.64 (d, *J* = 6.4 Hz, 2H), 3.84 (s, 3H). ^{13}C NMR (101 MHz, DMSO-d_6) δ 166.58, 164.56, 162.29, 151.96, 147.68, 145.62, 135.54, 133.26, 131.29, 129.98, 129.73, 129.49, 129.11, 129.00, 128.63, 128.01, 122.23, 121.86, 119.23, 114.18, 99.74, 69.77, 52.53, 42.90. HRMS (ESI$^+$): *m/z* calculated for $C_{28}H_{25}N_3O_5$ 484.1872, found $[M + H]^+$ 484.1894.

3.61 (*E*)-*N*-(4-(3-(hydroxyamino)-3-oxoprop-1-en-1-yl)benzyl)-4-phenethoxyquinoline-2-carboxamide (SDFZ-15)

11g (0.23 g, 0.50 mmol) was dissolved in 5 mL potassium hydroxide, hydroxylamine methanol solution and 5 mL DMF, then were treated according to **SDFZ-1** to give **SDFZ-15** (0.03 g) as white solid, yield: 15%, melting point:150 - 152 ℃。^{1}H NMR (400 MHz, DMSO-d_6) δ 9.44 (s, 1H), 8.13 (d, *J* = 8.3 Hz, 1H), 8.04 (d, *J* = 8.5 Hz, 1H), 7.86 - 7.78 (m, 1H), 7.64 (d, *J* = 9.2 Hz, 2H), 7.51 - 7.22 (m, 10H), 6.45 (d, *J* = 15.8 Hz, 1H), 4.56 (s, 4H), 3.21 (t, *J* = 6.3 Hz, 2H). ^{13}C NMR (101 MHz, DMSO-d_6) δ 164.66, 162.51, 152.11, 147.62, 138.68, 131.14, 130.11, 129.53, 129.44, 128.83, 128.41, 128.26, 127.89, 127.81, 127.66, 127.27, 127.10, 126.87, 122.06, 121.78, 99.12, 69.70, 42.85, 35.06. HRMS (ESI$^+$): *m/z* calculated for $C_{28}H_{25}N_3O_4$ 468.1918, found $[M + H]^+$ 468.1894.

3.62 (*E*)-*N*-(3-(3-(hydroxyamino)-3-oxoprop-1-en-1-yl)benzyl)-4-methoxyquinoline-2-carboxamide (SDFZ-16)

11h (0.19 g, 0.50 mmol) was dissolved in 5 mL potassium hydroxide, hydroxylamine methanol solution and 5 mL DMF, then were treated according to **SDFZ-1** to give **SDFZ-16** (0.03 g) as white solid, yield: 18%, melting point:144 - 146 ℃. ^{1}H NMR (400 MHz, DMSO-d_6) δ 10.77 (s, 1H), 9.50 (t, *J* = 6.3 Hz, 1H), 9.05 (s, 1H), 8.20 (dd, *J* = 8.4, 1.4 Hz, 1H), 8.08 (d, *J* = 8.4 Hz, 1H), 7.85 (ddd, *J* = 8.4, 6.9, 1.5 Hz, 1H), 7.71 - 7.62 (m, 2H), 7.56 (s, 1H), 7.53 - 7.29 (m, 4H), 6.45 (d, *J* = 15.8 Hz, 1H), 4.58 (d, *J* = 6.4 Hz, 2H), 4.14 (s, 3H). ^{13}C NMR (101 MHz, DMSO-d_6) δ 164.73, 163.48, 163.05, 152.17, 147.59, 140.74, 138.57, 135.03, 131.18, 129.47, 129.22, 127.72, 126.89, 126.73, 122.10, 121.79, 119.60, 105.43, 98.65, 56.83, 42.98. HRMS (ESI$^+$): *m/z* calculated for $C_{21}H_{19}N_3O_4$ 378.1448, found $[M + H]^+$ 378.1414.

3.63 (*E*)-4-((4-(tert-butyl)benzyl)oxy)-*N*-(3-(3-(hydroxyamino)-3-oxoprop-1-en-1-yl)benzyl)quinoline-2-carboxamide (SDFZ-17)

11i (0.24 g, 0.50 mmol) was dissolved in 5 mL potassium hydroxide, hydroxylamine methanol solution and 5 mL DMF, then were treated according to **SDFZ-1** to give **SDFZ-17** (0.05 g) as white solid, yield: 25%, melting point: 260 - 262 °C. ^{1}H NMR (400 MHz, DMSO-d_6) δ 9.46 (s, 1H), 8.22 (d, *J* = 8.5 Hz, 1H), 8.07 (d, *J* = 8.1 Hz, 1H), 7.85 (s, 1H), 7.74 (s, 1H), 7.66 (s, 1H), 7.48 (dd, *J* = 5.6, 2.4 Hz, 5H), 7.36 (d, *J* = 8.6 Hz, 5H), 6.44 (d, *J* = 16.4 Hz, 1H), 5.46 (s, 2H), 4.55 (s, 2H), 1.30 (d, *J* = 2.9 Hz, 9H). ^{13}C NMR (101 MHz, DMSO-d_6) δ 172.97, 163.29, 152.30, 151.90, 147.58, 146.65, 145.62, 133.84, 133.45, 132.24, 131.09, 130.93, 129.20, 128.11, 127.47, 125.84, 122.51, 121.77, 117.11, 111.43, 99.67, 80.80, 78.05, 70.49, 68.63, 65.63, 34.83. HRMS (ESI$^+$): *m/z* calculated for $C_{31}H_{31}N_3O_4$ 510.2387, found [M + H]$^+$ 510.2385.

3.64 (*E*)-4-((4-chlorobenzyl)oxy)-*N*-(3-(3-(hydroxyamino)-3-oxoprop-1-en-1-yl)benzyl)quinoline-2-carboxamide (SDFZ-18)

11j (0.24 g, 0.50 mmol) was dissolved in 5 mL potassium hydroxide, hydroxylamine methanol solution and 5 mL DMF, then were treated according to **SDFZ-1** to give **SDFZ-18** (0.05 g) as white solid, yield: 22%, melting point: 148 - 150 °C. ^{1}H NMR (400 MHz, DMSO-d_6) δ 10.22 (s, 1H), 9.48 (s, 1H), 8.24 (d, *J* = 8.5 Hz, 1H), 8.08 (d, *J* = 8.4 Hz, 1H), 7.86 (t, *J* = 7.6 Hz, 1H), 7.76 - 7.60 (m, 5H), 7.52 (d, *J* = 7.7 Hz, 2H), 7.33 (t, *J* = 21.6 Hz, 4H), 6.46 (d, *J* = 15.8 Hz, 1H), 5.52 (d, *J* = 3.9 Hz, 2H), 4.57 (d, *J* = 6.4 Hz, 2H). ^{13}C NMR (101 MHz, DMSO-d_6) δ 164.62, 162.29, 152.80, 152.08, 147.67, 140.66, 138.59, 136.58, 136.13, 135.65, 133.26, 131.81, 131.23, 130.29, 130.04, 129.97, 129.48, 129.10, 127.81, 122.20, 121.83, 100.68, 99.87, 99.73, 69.79. HRMS (ESI$^+$): *m/z* calculated for $C_{27}H_{22}ClN_3O_4$ 488.1372, found [M + H]$^+$ 488.1393.

3.65 (*E*)-4-((4-fluorobenzyl)oxy)-*N*-(3-(3-(hydroxyamino)-3-oxoprop-1-en-1-yl)benzyl)quinoline-2-carboxamide (SDFZ-19)

11k (0.24 g, 0.50 mmol) was dissolved in 5 mL potassium hydroxide, hydroxylamine methanol solution and 5 mL DMF, then were treated according to **SDFZ-1** to give **SDFZ-19** (0.08 g) as white solid, yield: 35%, melting point: 160 - 162 °C. ^{1}H NMR (400 MHz, DMSO-d_6) δ 9.50 (t, *J* = 6.4 Hz, 1H), 8.23 (d, *J* = 8.3 Hz, 1H), 8.09 (d, *J* = 8.6 Hz, 1H), 7.88 - 7.83 (m, 1H), 7.76 (s, 1H), 7.72 - 7.55 (m, 5H), 7.44 - 7.35 (m, 2H), 7.29 (dd, *J* = 10.0, 7.7 Hz, 2H), 6.50 (d, *J* = 16.0 Hz, 1H), 5.50 (s, 2H), 4.59 (d, *J* = 6.5 Hz, 2H). ^{13}C NMR (101 MHz, DMSO-d_6) δ 167.92, 163.72, 163.65, 163.22, 161.29, 151.35, 144.27, 140.52, 134.68, 132.49, 131.85, 131.60, 131.51,

130.91, 130.75, 130.69, 130.60, 130.54, 129.86, 129.45, 128.10, 127.72, 127.32, 127.16, 122.37, 121.77, 121.51, 119.78, 116.07, 115.86, 100.07, 70.48. HRMS (ESI^+): *m/z* calculated for $C_{27}H_{22}FN_3O_4$ 472.1667, found $[M + H]^+$ 472.1667.

3.66 (*E*)-*N*-(3-(3-(hydroxyamino)-3-oxoprop-1-en-1-yl)benzyl)-4-phenethoxyquinoline-2-carboxamide (SDFZ-20)

11l (0.23 g, 0.50 mmol) was dissolved in 5 mL potassium hydroxide, hydroxylamine methanol solution and 5 mL DMF, then were treated according to **SDFZ-1** to give **SDFZ-20** (0.07 g) as white solid, yield: 32%, melting point: 150 - 152 °C. ^{1}H NMR (400 MHz, DMSO-*d*$_6$) δ 9.52 (t, *J* = 6.5 Hz, 1H), 8.18 - 8.12 (m, 1H), 8.06 (d, *J* = 8.4 Hz, 1H), 7.84 (ddd, *J* = 8.4, 6.8, 1.4 Hz, 1H), 7.70 - 7.61 (m, 2H), 7.57 (d, *J* = 1.9 Hz, 1H), 7.55 - 7.15 (m, 9H), 4.65 - 4.50 (m, 4H), 3.22 (t, *J* = 6.4 Hz, 2H). ^{13}C NMR (201 MHz, DMSO-*d*$_6$) δ 164.68, 162.51, 152.11, 147.62, 138.69, 133.88, 131.16, 129.54, 129.44, 128.84, 128.42, 127.89, 127.67, 126.88, 122.06, 121.78, 119.33, 99.13, 69.71. HRMS (ESI^+): *m/z* calculated for $C_{21}H_{19}N_3O_4$ 468.1918, found $[M + H]^+$ 468.1899.

3.67 (*E*)-*N*-(3-(3-(hydroxyamino)-3-oxoprop-1-en-1-yl)benzyl)-4-(naphthalen-1-ylmethoxy)quinoline-2-carboxamide (SDFZ-21)

11m (0.25 g, 0.50 mmol) was dissolved in 5 mL potassium hydroxide, hydroxylamine methanol solution and 5 mL DMF, then were treated according to **SDFZ-1** to give **SDFZ-21** (0.07 g) as brown solid, yield: 28%, melting point: 206 - 208 °C. ^{1}H NMR (400 MHz, DMSO-*d*$_6$) δ 10.78 (s, 1H), 9.51 (s, 1H), 9.11 (s, 1H), 8.34 - 8.19 (s, 1H), 8.09 (s, 1H), 7.98 - 7.15 (m, 12H), 6.46 (d, *J* = 15.3 Hz, 1H), 5.52 (s, 2H), 4.58 (s, 2H). ^{13}C NMR (101 MHz, DMSO) δ 152.15, 140.77, 135.60, 131.28, 130.06, 130.01, 129.51, 129.21, 129.14, 122.26, 122.24, 99.79, 69.87, 69.83. HRMS (ESI^+): *m/z* calculated for $C_{27}H_{23}N_3O_4$ 454.1761, found $[M + H]^+$ 454.1771.

References

1. Manfredini S, *et al.* (2002) Design, synthesis and activity of ascorbic acid prodrugs of nipecotic, kynurenic and diclophenamic acids, liable to increase neurotropic activity. *J. Med. Chem.* 45(3):559-562.

2. Liang T, *et al.* (2020) HDAC-Bax Multiple Ligands Enhance Bax-Dependent Apoptosis in HeLa Cells. *J. Med. Chem.* 12083-99.

3. Zhou Y, *et al.* (2017) Design, synthesis, and preliminary bioactivity evaluation of N-benzylpyrimidin-2-amine derivatives as novel histone deacetylase inhibitor. *Chem. Biol. Drug. Des.* 90(5):936-942.

4. Chen C, Yang XY, Fang H, & Hou XB (2019) Design, synthesis and preliminary bioactivity evaluations of 8-hydroxyquinoline derivatives as matrix metalloproteinase (MMP) inhibitors. *Eur. J. Med. Chem.* 181.

5. Zhou Y, *et al.* (2020) Discovery of Peptide Boronate Derivatives as Histone Deacetylase and Proteasome Dual Inhibitors for Overcoming Bortezomib Resistance of Multiple Myeloma. *J. Med. Chem.* 63(9):4701-4715.

6. Choudhary G & Peddinti RK (2011) Introduction of a clean and promising protocol for the synthesis of β-amino-acrylates and 1,4-benzoheterocycles: an emerging innovation. *Cheminform* 13(11):3290-3299.

7. Zhou Y, *et al.* (2012) Substituent effect of ancillary ligands on the luminescence of bis[4,6-(di-fluorophenyl)-pyridinato-N,C2']iridium(III) complexes. *Dalton Trans* 41(31):9373-9381.

8. Francesco G. Salituro BLHBMBPLNKTSJH (1992) 3-(2-Carboxyindol-3-yl)propionic acid-based antagonists of the N-methyl-D-aspartic acid receptor associated glycine binding site. *Journal of Medicinal Chemistry* 35(10):1791.

9. Manfredini S, *et al.* (2004) Design, synthesis and in vitro evaluation on HRPE cells of ascorbic and 6-bromoascorbic acid conjugates with neuroactive molecules. *Bioorg Med Chem* 12(20):5453-5463.
